# Evaluation of photoinhibition and development of stress tolerance index to identify photosynthetically adapted and stress tolerant germplasm accessions of tepary bean

**DOI:** 10.1371/journal.pone.0357919

**Published:** 2026-09-11

**Authors:** Juan Carlos Suárez, Amara Tatiana Contreras, Idupulapati M. Rao

**Affiliations:** 1 Programa de Ingeniería Agroecológica, Facultad de Ingeniería, Universidad de la Amazonia, Florencia, Colombia; 2 Programa de Maestría en Bioestadística, Facultad de Ingeniería, Universidad de la Amazonia, Florencia, Colombia; 3 Grupo de Investigaciones Agroecosistemas y Conservación en Bosques Amazónicos-GAIA, Centro de Investigaciones Amazónicas CIMAZ Macagual César Augusto Estrada González, Florencia, Colombia; 4 Programa de Maestría en Sistemas Sostenibles de Producción, Facultad de Ciencias Agropecuarias, Universidad de la Amazonia, Florencia, Colombia; 5 International Center for Tropical Agriculture (CIAT), Cali, Colombia; UTRGV: The University of Texas Rio Grande Valley, UNITED STATES OF AMERICA

## Abstract

Knowledge about photosynthetic adaptation and stress tolerance in tepary bean (*Phaseolus acutifolius*) accessions under acidic soil and high‑temperature conditions in the Colombian Amazon is limited. We developed a stress tolerance index (STI) as a helpful scorecard based on physiological measurements of 323 accessions that were grown in acidic soil under field and screenhouse conditions and were evaluated under well‑watered and water‑stressed treatments at dawn and midday. Based on STI and complementary photosynthetic traits, accessions were classified into five adaptive categories (highly: n = 34, 10.5%; moderately: n = 49, 15.1%; slightly: n = 79, 24.4%; poorly: n = 100, 30.9%; very poorly: n = 61, 18.8%). Three accessions (G40068, G40302, G40087) stood out in overall performance: G40087 had the highest STI (0.754), G40068 the highest F_v_/F_m_ (0.737), and G40302 the highest qL (0.562). These accessions also exhibited higher relative chlorophyll content (between 45 and 52), greater leaf thickness (between 280 and 320 μm), a maximum photochemical efficiency of about 0.73, and an effective quantum yield of >0.45 under stress—traits that supported better photosynthetic performance. These materials could serve as promising parents for breeding climate‑resilient beans adaptive to the western Amazon, but their agronomic value requires validation in yield and biomass trials under the same stress conditions.

## 1. Introduction

The global ambient temperature is expected to rise by 1.5 °C [1], a situation that implies a longer duration and increased frequency of heat stress episodes, thereby heightening the risk of drought [2,3], which could lead to a reduction in crop yields by 10–17% [3,4]. These gradual temperature increases, especially in tropical and subtropical regions, pose significant challenges for production of food crops [5,6]. Developing countries located in Africa, Asia, and Latin America, situated in these two regions, will suffer even more from these effects due to their limited resources to cope with changing conditions [7]. Consequently, over the past decade, many studies have focused on plant responses to a single stress factor [8–15], which provide an insight into different adaptive strategies used by crops to maintain their productivity [16]. However, under field conditions, plants are subjected to a simultaneous combination of different abiotic stresses [17], resulting in a response that cannot be extrapolated from the plant’s response to each stress applied individually [18,19]. When two stress conditions occur simultaneously, the plant’s adaptive strategy to the combination of stresses is governed by the interaction of the two stresses, which the plants perceive as a new state of stress [18].

Acidic soils and high temperatures are two critical threats to crop productivity [14,15]. The combined effect of these two stresses alters various agronomic characteristics by affecting biochemical and physiological functions [16], leading to impacts on plant growth, development, and yield [20]. Under acidic soil stress, plants experience physiological disruptions due to aluminum toxicity and phosphorus deficiency, particularly in the partitioning of photosynthates and metabolic activities, resulting in yield loss [17,21,22]. Similarly, heat stress affects metabolic activities such as the inactivation of enzymatic activities, protein synthesis, cell membrane damage, and impacts on the biochemical reactions of photosystem II (PSII) [16,17]. The incidence of both stresses at the physiological level is reflected in the efficiency of light energy utilization and the reduction of photosynthetic machinery, specifically in the quantum yield (F_v_/F_m_) and PSII activity [20]. These effects are positively associated with the generation of reactive oxygen species (ROS) [6], which can result in photoinhibition and photodamage of PSII depending on the plants’ strategies to adjust and recover their photosynthetic function [3,23].

Among the grain legume species, the common bean (*Phaseolus vulgaris* L.) is one of the most cultivated, and it is considered a vital nutritional source for global food security [24,25]. However, under the current environmental conditions with warmer and/or drier climates due to climate change, production areas worldwide have been and will continue to be rapidly reduced [26–28]. To address these limitations, new bean varieties resistant to combinations of different stresses must be developed using genetic resources of stress resistance from different *Phaseolus* species [29,30]. Genetic resources of closely related *Phaseolus* species can leverage natural variation for adaptation to abiotic stresses such as heat and drought [31]. As a sister species to common beans, the tepary bean (*Phaseolus acutifolius* A. Gray) has gained attention in breeding programs, mainly as a gene donor to improve abiotic stress tolerance in common bean [15]. Being cultivated in desert and semi-arid regions makes it a valuable crop for dry and high-temperature environments [32]. Compared to the common bean, the tepary bean exhibits different stress tolerance mechanisms, including deep rooting to avoid dehydration [33] and increased leaf production to compensate for reduced leaf size due to heat stress [34]. It also exhibits lower sensitivity of the mitochondrial electron transport metabolism and better stomatal control to reduce water use [32], and these are important attributes for improving the adaptation of common beans to dry and hot environments.

In the western Amazonian conditions, previous research was focused on understanding the physiological characteristics of the cultivated tepary bean and its wild relatives [35]. These studies revealed morpho-phenological and agronomic attributes of cultivated *P. acutifolius* var. acutifolius with more pods per plant, larger seeds, and a higher seed count per pod. In terms of energy dissipation mechanisms, photochemical quenching (qP) was greater in cultivated var. acutifolius accessions, whereas non-photochemical quenching (NPQ) was more pronounced in regressive var. acutifolius and wild var. tenuifolius accessions. Furthermore, Suárez and Rao [36] identified significant differences in photosynthetic performance under heat stress, particularly in F_v_/F_m_ and Φ_2_ variables, which reflect differences in quantum yield and photosystem II function. Some accessions demonstrated efficient leaf cooling capabilities without increased water use, enhancing heat tolerance. These studies were conducted under natural environmental conditions, avoiding maximum stress levels. There is a need to test the recovery capacity of tepary bean accessions under higher temperature that can be induced under screenhouse conditions compared to field conditions during periods of maximum and minimum rainfall in the western Amazon region.

Although there are detailed studies comparing the wild and cultivated ancestors of the common bean, there is limited information on the adaptive responses of the tepary bean to more humid conditions in warmer tropical regions of Central America, the Andes, and the Amazon basin [34,35,37,38]. Obtaining information on phenotypic and genotypic attributes becomes an important resource for selecting new progenitors with potential and desirable characteristics to develop varieties adapted and tolerant to variability of climate [7]. Selecting tepary bean accessions resistant to combined stresses requires careful work, as either tolerance or resistance to adverse situations involves a complex network of physiological and genetic mechanisms influenced by different environmental conditions [14]. At the physiological level, adaptation to high temperatures and acid soil stress could be assessed through the use of chlorophyll fluorescence (Chl_a_) measurements. These measurements help quantify genotypic differences in energy use in response to stress levels, particularly the energy absorbed by PSII distributed to photochemical, heat dissipation, or unregulated pathways (i.e., energy going to photochemistry ΦII, regulated heat dissipation ΦNPQ (non-photochemical quenching), and non-regulated energy loss ΦNO) and leaf cooling [35]. This energy partitioning is a vital protective mechanism against excess light, which can damage the photosynthetic apparatus. The present study highlights significant results regarding the physiological behavior of different tepary bean accessions within a screenhouse setting and under environmental conditions where the temperature was elevated by 1.5 °C above ambient levels (37 °C) during the period of minimal precipitation in the Colombian Amazon. The main objective of this study was to determine the differences in physiological behavior of tepary bean germplasm accessions grown under two treatments (well-watered and water-stressed) and measured at two times during the day (dawn and midday) on plants grown under two different growing conditions (field and screenhouse) in the Colombian Amazon. The specific hypothesis tested was that there are significant differences in photosynthetic responses among tepary bean germplasm accessions, and these differences contribute toward their overall level of stress tolerance to the combined stress conditions of acid soil and high temperature.

## 2. Materials and methods

### 2.1. Experimental site and meteorological conditions

**Fig 1:**
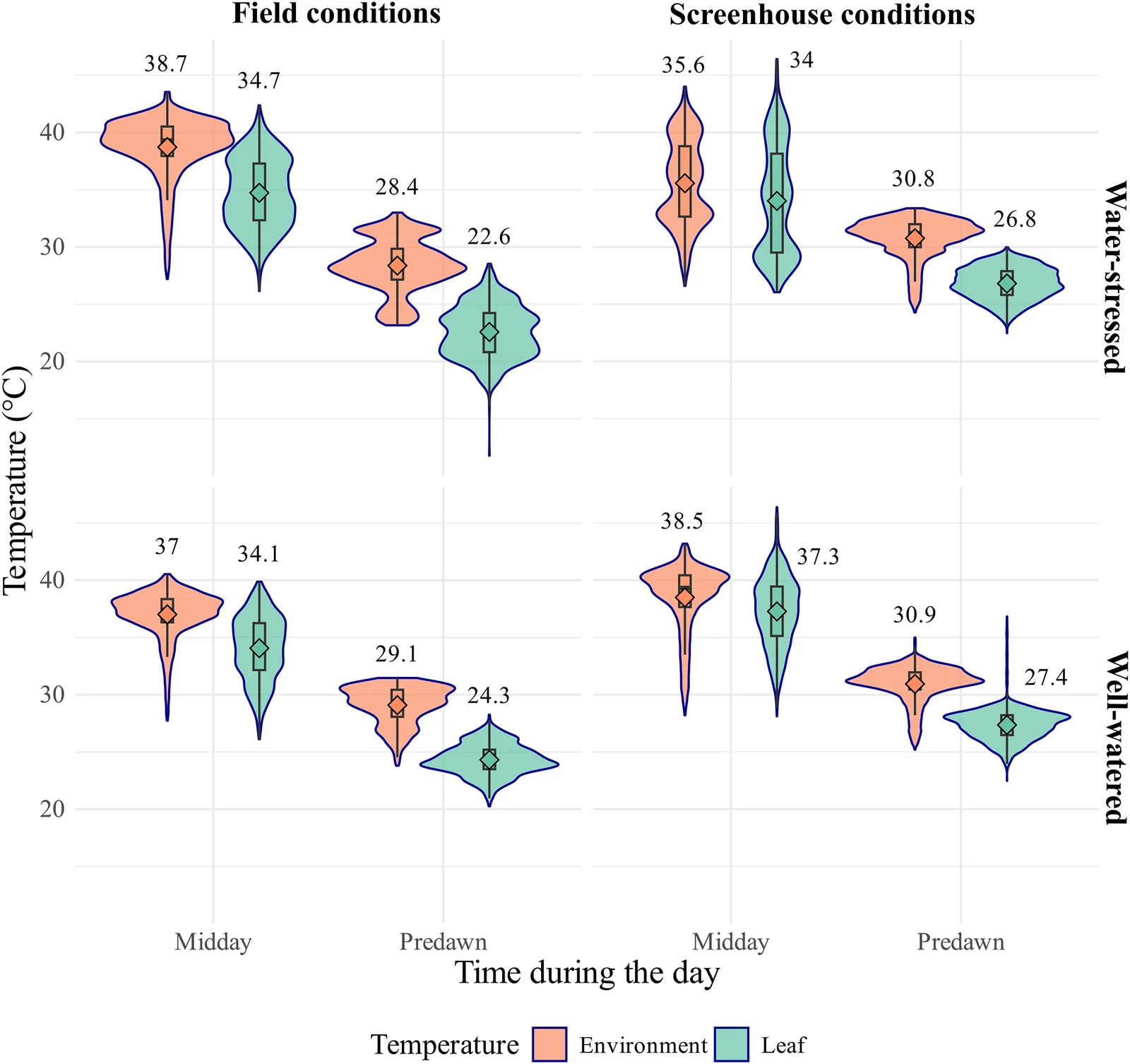
Daily variation in ambient and leaf temperature in different accessions of tepary bean under field and screenhouse conditions during periods of water-stressed and well-watered at the CIMAZ Macagual Amazon Research Center of the University of Amazonia.

At the Centro de Investigaciones Amazónicas CIMAZ Macagual (1°37′ N and 75°36′ W) of the Universidad de la Amazonia, located in Florencia, Caquetá (Colombia), an evaluation of 323 germplasm accessions of tepary bean (*Phaseolus acutifolius*) was carried out in the field and screenhouse in pot-grown plants under combined stress conditions of acid soil and high temperature. This research center is located within a tropical rainforest ecosystem and is characterized by a mean annual temperature of 25.5 °C, a relative humidity of 84%, a mean annual rainfall of 3,800 mm and 1,700 hours of sunshine per year. Evaluations of the physiological performance of the tepary bean accessions were carried out during two seasons and corresponded to the following periods: i. from December 2022 to March 2023, and ii. from February 2023 to July 2023. Fig 1 shows how variations were presented between field and screenhouse growing conditions, affecting both the ambient temperature and the leaf temperature of the tepary bean accessions. Overall, thermal variations were more pronounced under screenhouse conditions than in the field, with a mean relative increase of 8.6% for water‑stressed plants and a smaller net variation of 2.3% for well‑watered plants (derived from the mean of the four comparisons between midday/predawn and leaf/ambient values across screenhouse and field). Concerning diel timing, temperatures were markedly higher at midday versus predawn: in well‑watered plants, ambient temperature rose from 28.4 °C (predawn) to 39.7 °C (midday), corresponding to a + 39.8% increase, while leaf temperature increased from 22.6 °C to 34.1 °C, a + 50.9% increase. Under water-stressed conditions, ambient temperature increased from 29.1 °C (predawn) to 37.0 °C (midday), a + 27.1% change, and leaf temperature increased from 21.8 °C to 34.4 °C, representing a + 57.8% change. These differences reflect two combined effects: (i) the screenhouse tends to attenuate the diurnal ambient temperature peak in well‑watered plants while augmenting nocturnal heat retention, and (ii) under water stress conditions the screenhouse amplifies canopy warming both at predawn and midday, implying reduced transpiration‑mediated cooling capacity and greater heat accumulation within the canopy.

### 2.2. Plant material and experimental design

Seeds of a total of 323 germplasm accessions of tepary bean (*Phaseolus acutifolius*) were provided from the Future Seeds (genebank) of the Alliance of Bioversity International and International Center for Tropical Agriculture (CIAT) [39]. This tepary bean germplasm collection is composed of cultivated, regressive, and wild materials that have shown variation in tolerance to acidic soils and high temperatures under the conditions of the Colombian Amazon [35]. In each evaluation period, the tepary bean accessions were simultaneously planted both under field conditions and in a screenhouse using 12-liter volume pots. Soil used to grow plants was characterized by very low base saturation of around 7.1% (cmol kg^−1^: Ca: 0.38; Mg: 0.1; K: 0.14; Na: 0.1) and a cation exchange capacity of 11.3 cmol kg^−1^, with a pH ranging between 4.1 and 5.2. Additionally, it had low soil organic matter content (1.35%), and low available phosphorus (2.58 mg kg^-1^, Bray-II) with high level of exchangeable aluminum (6.3 cmol(+) kg^−1^), classifying it as aluminum-toxic acidic soil with low fertility. The soil used in the experiment received neither amendments to correct acidity nor application of synthetic fertilization to improve its fertility. To evaluate photosynthetic adaptation and stress tolerance of the tepary bean accessions under combined stress conditions of acidic soil and high temperature, a completely randomized plot design with a factorial arrangement was used (i. tepary bean accessions, ii. plant growth conditions (field conditions, screenhouse conditions), iii. water supply level (well-watered, water-stressed)) where each plot corresponded to each tepary bean accession. Ten pots were used in each plot, and each contained one plant for a total of 12,920 plants monitored (323 tepary bean accessions × 10 pots × 2 plant growth condition × 2 levels of water supply) for adaptive responses.

### 2.3. Physiological behavior of tepary bean accessions under combined stress conditions of acid soils and high temperatures

Sampling was carried out during the two periods of water availability to plants in each temporal repetition, where: i. water-stressed conditions, defined as a period of at least 15 days without rainfall events, where the soil moisture level ranged from 10 to 14% and in ii. well-watered conditions, characterized by the average accumulation of more than 130 mm in a 15-day period reaching a soil moisture level of 22–28%. Soil moisture measured using a 5TE sensor (METER Group, Inc., Pullman, WA, USA). It should be noted that this variation in water availability to plants was simulated by manual irrigation under the screenhouse conditions, so that each rainfall event that occurred under natural field conditions was reproduced in the screenhouse to ensure the consistency of the soil water limitation conditions used in this study. A WatchDog 2900ET weather station (Spectrum Technologies, Inc., USA) was used to monitor precipitation events throughout the experiment. This information was used to simulate irrigation for the plants growing under screenhouse conditions. In each water‑availability period (water-stressed and well‑watered), one fully expanded leaf from the upper canopy—located between the seventh and ninth leaf from the plant base—was selected from three plants per tepary bean accession. Measurements were taken during flowering time (R_6_; ~ 50% of flowers open), approximately 40 days after sowing according to the BBCH scale (BBCH 65), and sampling was repeated for three consecutive days. On the first day, sampling was performed only at midday (11:30–14:00 hours), since the data collected at predawn (04:00–05:30 hours) on the second day will determine the level of photoinhibition [23], a process that was repeated for three consecutive days with the objective of having temporal repetitions at the time level (i. midday, ii. predawn) during the day.

Fluorescence and absorbance-based photosynthetic parameters were determined using ten portable field spectrophotometers (MultispeQ V2.0 devices, PhotosynQ INC., East Lansing, Michigan, USA) simultaneously operated under both field and screenhouse conditions, with all 10 devices connected to the PhotosynQ platform (http://www.photosynq.org) during data acquisition. The MultispecQ v2.0 device was connected via Bluetooth to the PhotosynQ app for Android using a smartphone (https://www.photosynq.com/software). Before each sampling, the device was calibrated using the CaliQ system (PhotosynQ INC, East Lansing, Michigan, USA). This device records different environmental variables and the performance of both PSII and PSI using chlorophyll fluorescence parameters. The data collected from the tepary bean accessions include two periods of water availability (water-stressed and well-watered) under both field and screenhouse conditions, at predawn and midday periods and the data were taken during three consecutive days, corresponding to a total of 16,992 data points, and all this information is available in PhotosynQ [40,41].

The Photosynthesis RIDES 2.0_no_open_close protocol was used, which is designed to collect data on photosynthetic efficiency and other physiological aspects of plants related mainly to chlorophyll fluorescence, ATP synthase conductance, estimation of the saturation pulse of PSI parameters, as well as the turnover rate of the cytochrome *b*_*6*_*f* complex through DIRK_P700_. This protocol is designed for measurements on light‑adapted leaves and does not require prior dark adaptation; fluorescence measurements are made on the light‑adaptive state and F_m_’ is obtained using saturation pulses integrated into the sequence. The measuring light is the very low‑intensity modulated light produced by the MultispeQ LEDs, designed to minimize photosynthetic perturbation (equivalent to a very low photosynthetic flux, on the order of <5 µmol·m^−2^·s^−1^ for comparison), while the actinic light used reached 8,000 µmol·m^−2^·s^−1^. This level is typically used for strong light-acclimation curves, high-light stress assays, or saturating photosynthesis in sun-adapted leaves. Predawn measurements (04:00–05:30 h) were also taken and provide ≥4 h natural dark adaptation used to determine F_v_/F_m_ and photoinhibition indices. Dark-Interval Relaxation Kinetics (DIRK) analysis technique is used to measure the redox state and electron turnover rate of P700, a crucial pigment in photosystem I (PSI) during photosynthesis. By observing the rapid reduction of oxidized P700 (P700+) after a dark interval, it is possible to calculate the rate of electron flow through PSI, which helps in understanding photosynthetic capacity, cyclic electron transport, and protection against oxidative stress.

Using the MultispeQ device, the ambient temperature (T_a_), relative humidity (RH) and radiation level (photosynthetically active radiation, PAR) were measured by means of the different sensors built into the MultispeQ. Likewise, the leaf temperature (LT) was determined and leaf temperature difference (LTD = T_a_ - LT) was determined. The different chlorophyll fluorescence variables (F_o_ = minimum fluorescence, F_m_ = maximum fluorescence, F_s_ = steady state fluorescence and F_v_/F_m_ = PSII quantum efficiency) were used to determine the energy pathway (ΦII = quantum yield of PSII electron transport, ΦNO = unregulated energy, ΦNPQ = energy dissipated as heat, [42], NPQ_t_ = total non-photochemical quenching of absorbed light energy [43], qL = fraction of open PSII reaction centers [44], LEF = linear electron flow [45]). The rate (vH+) was measured as the proton conductance of ATP synthases in the thylakoid membrane (gH+), which determines the function of ATP synthase, as well as the maximum electrochromic displacement amplitude (ECS_t_) [46]. Following the protocols proposed by Kanazawa et al. [47], the different states of photosystem I (PSI) reaction centers: i. active (PSI_act_), ii. oxidized (PSI_ox_), iii. open state (PSI_open_), and iv. over-reduced (PSI_or_) were measured. In addition, different parameters were calculated which were related to the constant with which the electron transfer is performed at P700 (_k_P700), the initial velocity of electron transfer at P700 in steady state (_i_P700), the electron transfer lifetime of P700 (_t_P700), and the electron transfer amplitude of P700 during the light-dark transition (P700_DIRK_), which indicates the ability of PSI to transfer electrons [47]. In addition, leaf thickness (LTh) in micrometers [44] and relative chlorophyll (RC) content were also measured (S1 File).

### 2.4. Determination of the level of stress caused by acid soils and high temperatures in tepary bean accessions

The level of stress caused by the combined abiotic stress factors of acid soil and high temperature was measured using PSII photoinhibition indices including both chronic photoinhibition (PI_Chr_) and dynamic photoinhibition (PI_Dyn_) which were calculated with data obtained at predawn and midday PSII photochemical efficiency (F_v_/F_m_), according to Werner et al. [23]. These indices show the plant’s capacity to generate regulatory processes related to the dissipation of excess energy under stress conditions [48] and these have been used to measure the level of tolerance to high temperature and radiation stress [28,49]. In this sense, PI_Chr_ corresponds to the effect of prolonged exposure to the stress condition and is slowly reversible, the functioning of the photosynthetic apparatus. However, PI_dyn_ shows the capacity of recovery of the photosynthetic apparatus to the stress exposure that occurs during the day but can be recovered at night. These indices were calculated as follows:

-
${\text{PI}}_{\text{Chr}}=\left[{\left({\text{F}}_{\text{y}}/{\text{F}}_{\text{m}}\right)}_{\text{max}}-{\left({\text{F}}_{\text{y}}/{\text{F}}_{\text{m}}\right)}_{\text{pd}}\right]/{\left({\text{F}}_{\text{y}}/{\text{F}}_{\text{m}}\right)}_{\text{max}}\times \text{100}$

-
${\text{PI}}_{\text{Dyn}}=\left[{\left({\text{F}}_{\text{y}}/{\text{F}}_{\text{m}}\right)}_{\text{pd}}-{\left({\text{F}}_{\text{y}}/{\text{F}}_{\text{m}}\right)}_{\text{mid}}\right]/{\left({\text{F}}_{\text{y}}/{\text{F}}_{\text{m}}\right)}_{\text{max}}\times \text{100}$

-
${\text{PI}}_{\text{Total}}={\text{PI}}_{\text{Chr}}+{\text{PI}}_{\text{Dyn}}$

where: a. chronic photoinhibition (PI_Chr_), shows the percentage value by which F_v_/F_m_ is reduced in each condition with respect to the maximum F_v_/F_m_ reached during monitoring (pd: Predawn, mid: Midday); b. dynamic photoinhibition (PI_Dyn_), determines the percentage value by which F_v_/F_m_ is modified in relation to that obtained at noon on the previous day with respect to the value obtained at dawn of the following day, in relation to the maximum F_v_/F_m_ value observed during the entire experiment; and c. total photoinhibition (PI_Total_).

Additionally, a synthetic indicator called Stress Tolerance Index (STI) as a helpful scorecard was generated, based on the calculations proposed by Ramón et al. [50], and was adjusted with the objective of eliminating the intra- and inter-accession variability with respect to the median value of the population of tepary bean accessions in each evaluation period following the procedures proposed by Suárez et al. [35]. In the first instance, the STI was obtained by determining the relationship of all the variables obtained using MultispeQ, which are compiled in a general way based on the physiological responses observed under different conditions. These include the variables directly related to the functioning of the photosynthetic apparatus (F_v_/F_m_) and the fraction of energy effectively dedicated to the photosynthetic machinery (ΦII). With the correlations and by transforming the variables between a range of 0 (minimum) to 1 (maximum) and considering the criterion of the variables that when i) More is better, that is, the higher the physiological variable is, the better the plant is performing adequately with values close to one, and when ii) Less is better, referring to those variables that are closer to zero because the plant is performing adequately at the physiological level, the STI was obtained in an additive manner. Based on the above strategy, the STI explains which genotype is tolerant or not as it is based on the principle of transformation of variables in the range of 0–1 value where a higher value is given to variables that contribute positively and negatively to the overall physiological functioning. The following describes the normalization process for variable k for accession j in period p:

*If “more is better”:*


$$\begin{array}{c} {\text{norm}}_{j,p,k}=\frac{{\text{data}}_{j,p,k}-V_{\text{min},p,k}}{V_{\text{max},p,k}-V_{\text{min},p,k}} \end{array}$$

Where:

${\text{norm}}_{j,p,k}$: Normalized value for accession j, period p, and variable k.

${\text{data}}_{j,p,k}$: Actual data value for accession j, period p, and variable k.

$V_{\text{min},p,k}$: Minimum value for variable k in period p.

$V_{\text{max},p,k}$: Maximum value for variable k in period p.

This formula scales the data such that the minimum value becomes 0 and the maximum value becomes 1, assuming higher values are better.

*If “less is better”:*


$$\begin{array}{c} {\text{norm}}_{j,p,k}=\frac{V_{\text{max},p,k}-{\text{data}}_{j,p,k}}{V_{\text{max},p,k}-V_{\text{min},p,k}} \end{array}$$

This process has the same components as the previous ones. This formula scales the data so that the maximum value becomes 0 and the minimum value becomes 1, assuming that lower values are better.

Once all variables have been normalized, the Initial STI (average of the K normalized variables) for accession j in period p:


$$\begin{array}{c} {\text{STIinit}}_{j,p}=\frac{\text{1}}{K}\cdot \sum_{k=\text{1}}^{K}{\text{norm}}_{j,p,k} \end{array}$$

Where:

${\text{STIinit}}_{j,p}$: Initial Stress Tolerance Index for accession j in period p.

K: Total number of variables considered.

$\sum_{k=\text{1}}^{K}{\text{norm}}_{j,p,k}$: Sum of normalized values for all variables k.

This calculates the average normalized value across all variables for a given accession and period.

Definition of the photosynthetic index Y (using initial STI as Y):


$$\begin{array}{c} Y_{j,p}={\text{STIinit}}_{j,p} \end{array}$$

Where:

$Y_{j,p}$: Photosynthetic index for accession j in period p.

Directly uses the initial STI as the photosynthetic index.

Geometric mean is used of the experiment in period p:


$$\begin{array}{c} {\bar{Y}}_{p}={\left(\prod_{j=\text{1}}^{J}\text{max}(Y_{j,p},\varepsilon )\right)}^{\frac{\text{1}}{J}} \end{array}$$

Where:

${\bar{Y}}_{p}$: Geometric mean of the photosynthetic indices for period p.

J: Total number of accessions.

$\prod_{j=\text{1}}^{J}\text{max}(Y_{j,p},\varepsilon )$: Product of the photosynthetic indices, using ε to avoid zeros.

The geometric mean is used to aggregate performance across multiple accessions.

Reduction of the photosynthetic apparatus performance (RPaP) — relative value (%):


$$\begin{array}{c} {\text{RPaP}}_{j,p}=\left(\frac{Y_{j,p}}{{\bar{Y}}_{p}}\right)\cdot \text{1}\text{0}\text{0} \end{array}$$

Where:

${\text{RPaP}}_{j,p}$: Relative performance reduction for accession j in period p.

Compares the photosynthetic index of a specific accession to the geometric mean, either as a percentage or a fraction.

General stress intensity index (SII) between period i and j (e.g., i=1 control, j=2 stress):


$$\begin{array}{c} \text{SII}=\text{1}-\left(\frac{{\bar{Y}}_{\text{period }\text{2}}}{{\bar{Y}}_{\text{period }\text{1}}}\right) \end{array}$$

Where:

SII: Stress Intensity Index.

Compares the geometric means of two periods to quantify the relative intensity of stress.

Geometric mean of the RPaP for accession X between the two periods:


$$\begin{array}{c} {\bar{Y}}_{\text{RPaP},X}={\left({\text{RPaP}}_{X,\text{1}}\cdot {\text{RPaP}}_{X,\text{2}}\right)}^{\frac{\text{1}}{\text{2}}} \end{array}$$

Where:

${\bar{Y}}_{\text{RPaP},X}$: Geometric mean of the RPaP for accession X.

Geometric mean is used to summarize performance across the two periods.

Final STI for accession X:


$$\begin{array}{c} {\text{STI}}_{X}=\frac{\text{1}-{\bar{Y}}_{\text{RPaP},X}}{\text{SII}} \end{array}$$

Where:

${\text{STI}}_{X}$: Final Stress Tolerance Index for accession X.

Combines the geometric mean of RPaP and the SII to assess overall stress tolerance. These formulas provide a comprehensive framework for evaluating the performance and stress tolerance of different accessions over varying periods.

### 2.5. Data analysis

To define five typologies of tepary bean germplasm accessions based on photosynthetic and photoinhibitory responses and tolerance to acidic soils and high temperatures, a rigorous statistical framework was employed to partition genetic variation from variation attributable to experimental conditions and repeated measures. First, adjusted means for each accession were estimated by fitting linear mixed models (LMMs) using the *lme* function of the *nlme* package [51] in R 4.5.1. [52]. In these models each physiological trait served as the response variable and “accession” was treated as a fixed effect to allow direct estimation of genotypic means of interest. Experimental factors that were not the primary objects of comparison were included as random effects to model dependency structure and variance heterogeneity: i. growth condition (field vs. screenhouse), ii. water supply level (well-watered conditions, water-stressed conditions), iii. time during the day (predawn, midday), iv. day of measurement (1, 2, 3, 4), and v. replication (1, 2, 3). Assumptions were checked systematically: residual normality was assessed with Q–Q plots and complementary visual diagnostics; homoscedasticity was examined via residuals versus fitted-value plots and by comparing variances across factor levels. Where evidence of heterogeneity of variances was present, different variances by level were modeled using *varIdent* (nlme), allowing variance heterogeneity for relevant factors (e.g., growth condition or water supply level). Cluster analysis was applied to the LMM-adjusted means to identify typologies, followed by principal component analysis (PCA) to characterize multivariate relationships among typologies and physiological variables. The significance of variance explained by typologies in multivariate space was assessed by a Monte Carlo test with 999 permutations. For mean comparisons among typologies, an analogous LMM was fitted with “typology” as a fixed effect and “accession” as a random effect; post-hoc contrasts were adjusted using the DGC test at a 5% error level. Bivariate relationships were evaluated with Pearson correlations, and visualizations included frequency distributions, quadrant scatterplots with marginal means on the axes, and correlation matrices; plots were produced using *ade4*, *ggplot2*, *factoextra* and *corrplot*. At every step, criteria for selecting covariance structures were documented and model diagnostics and AIC/BIC criteria supporting modeling decisions were reported. This integrative approach—LMMs to adjust for design effects and obtain adjusted means, explicit correction for heterogeneity via *varIdent*, multivariate classification, and permutation tests for significance—ensured that inferences about differences among accessions and typologies reflected genetic variation of interest rather than artifacts arising from experimental structure or multiple testing.

Sensitivity analysis was performed to evaluate how STI varies in relation to each of the physiological variables used in its computation. The main purpose of this analysis was to determine the percentage-level variation of STI. We simulated scenarios in which each variable was decreased and increased by up to 100% and assessed the resulting impact on STI. Using the obtained information, we calculated the percent change in STI and its sensitivity to the different physiological variables by evaluating the slope of a linear regression model. Specifically, for each predictor variable we proceeded as follows: first, a simple linear regression of the form STI ~ X (where X is the physiological variable of interest) was fitted using only complete cases for STI and X. From the estimated slope (β1) and intercept (β0) of the fitted model, STI predictions were generated by applying percentage changes to the original value of X for each individual in the sample. The perturbations covered a range from −100% to +100% of the observed value in regular increments (e.g., 5%). For each observation, we computed X_mod = X_orig × (1 + pct/ 100) and evaluated the prediction STI_pred = β0 + β1 × X_mod.

From the observation-level predictions across percentage changes, we derived summary statistics for each perturbation level: the mean of STI_pred and its standard deviation. These summary curves allowed visualization of the aggregated effect of modifying each variable on the distribution of STI for each tepary accession. To quantify the overall effect magnitude, we also calculated the percent change in STI relative to its reference mean:


$$\begin{array}{c} \%\Delta \text{ STI}=\text{100}\times \left(\beta \text{1}\times \text{mean}(\text{X})\right)/\text{mean}(\text{STI})\text{.} \end{array}$$

This approximation expresses the relative variation in STI associated with a typical proportional change in X (the convention used was that the sign of %Δ follows the sign of β1). Individual figures display the prediction trajectories for each observation (spaghetti) and the mean prediction curve; additionally, a composite figure with all panels was compiled to facilitate comparison across variables. This procedure enabled identification of the variables to which STI is most sensitive, and quantification of the expected magnitude of STI change under relevant alterations in physiological conditions.

## 3. Results

### 3.1. Grouping of tepary bean accessions into five typologies based on physiological behavior to determine the level of tolerance to the combined stress of acid soil and high temperature

According to the cluster analysis of the different variables taken from the 323 tepary bean accessions, we found five statistically different typologies with contrasting photosynthetic mechanisms to tolerate high temperature stress. Based on photosynthetic adaptive traits, we divided the tepary bean accessions into the following five typologies: i. highly adaptive, ii. moderately adaptive, iii. slightly adaptive, iv. poorly adaptive, and v. very poorly adaptive (Table 1). The data presented represents the mean value of each variable from different accessions that are made up of each typology. The mean value for each tepary bean accession was obtained by correcting for the effect of different random factors such as i. plant growth conditions (field conditions, greenhouse conditions), ii. water supply level (well-watered conditions, water-stressed conditions), iii. time of day (predawn, midday), iv. day of measurement (1, 2, 3, 4), and v. replication (1, 2, 3). The typologies were generated based on differences in stress responsive traits (P < 0.0001). The variables that allowed the separation into five typologies were F_v_/F_m_, ΦII, ΦNPQ, NPQ_t_, and PI_Total_, variables that were related to the route taken by the energy and the level of photoinhibition experienced by each accession of tepary bean. The photosynthetic performance attributes of each typology are described below:

**Table 1:** Photosynthetic performance attributes evaluated among five different typologies of tepary bean accessions grown under acid soil and high temperature conditions in the Colombian Amazon.

Variable	Acronym	Highly adaptive	Moderately adaptive	Slightly adaptive	Poorly adaptive	Very poorly adaptive	Overall Mean	P-value
Relative chlorophyll content	RC	42.47 ± 1.32a	38.78 ± 0.87a	41.78 ± 1.1a	39.46 ± 0.77a	35.22 ± 0.99b	39.16 ± 0.44	<0.0001
Leaf temperature	LT	29.43 ± 0.19c	29.67 ± 0.12c	30.17 ± 0.16b	30.43 ± 0.11b	30.73 ± 0.14a	30.16 ± 0.07	<0.0001
Leaf temperature difference	LTD	−2.97 ± 0.18a	−3.33 ± 0.12a	−3.55 ± 0.15a	−3.38 ± 0.11a	−3.31 ± 0.13a	−3.34 ± 0.06	
Leaf thickness	LTh	0.46 ± 0.01a	0.45 ± 0.01a	0.44 ± 0.01a	0.42 ± 0.01b	0.42 ± 0.01b	0.43 ± 0.01	0.0006
Maximum electrochromic displacement amplitude (x 1000)	ECS_t_	0.55 ± 0.03a	0.57 ± 0.02a	0.63 ± 0.02a	0.64 ± 0.02a	0.61 ± 0.02a	0.63 ± 0.01	<0.0001
Proton conductance of ATP synthases in the thylakoid membrane	gH+	261.97 ± 20.04a	229.95 ± 13.15a	281.76 ± 16.69a	264.52 ± 11.69a	244.88 ± 14.96a	254.72 ± 6.54	
Rate of proton conductance of ATP synthases in the thylakoid membrane	vH+	0.12 ± 0.01a	0.11 ± 0.01b	0.13 ± 0.01a	0.13 ± 0.01a	0.12 ± 0.01a	0.12 ± 0.01	0.0011
Maximum fluorescence	F_m_	5635.58 ± 195.75a	5469.05 ± 128.42a	4688.2 ± 163.06b	4338.68 ± 114.14c	4104.11 ± 146.14c	4760.39 ± 71.01	<0.0001
Minimum fluorescence	F_o_	1491.55 ± 57.24a	1563.94 ± 37.55a	1257.05 ± 47.68b	1254.14 ± 33.38b	1256.47 ± 42.73b	1355.78 ± 20.34	<0.0001
Steady-state fluorescence	F_s_	2239.3 ± 86.21a	2285.41 ± 56.56a	1872.33 ± 71.81b	1860.79 ± 50.27b	1829.83 ± 64.36b	2000.39 ± 29.94	<0.0001
PSII photochemical efficiency	F_v_/F_m_	0.73 ± 0.01a	0.69 ± 0.01c	0.71 ± 0.01b	0.68 ± 0.01d	0.64 ± 0.01e	0.68 ± 0.01	<0.0001
Fraction of energy devoted to photosystem II	ΦII	0.56 ± 0.01a	0.52 ± 0.01c	0.55 ± 0.01b	0.51 ± 0.01d	0.47 ± 0.01e	0.52 ± 0.01	<0.0001
Linear electron flow	LEF	53.89 ± 1.41a	48.87 ± 0.93b	52.95 ± 1.18a	53.21 ± 0.82a	52.73 ± 1.06a	52.09 ± 0.47	0.0029
Fraction of open PSII reaction centers	qL	0.57 ± 0.01a	0.56 ± 0.01a	0.56 ± 0.01a	0.55 ± 0.01a	0.54 ± 0.01a	0.55 ± 0.01	
Total energy dissipated as heat	NPQ_t_	0.81 ± 0.04e	1.41 ± 0.03c	1.12 ± 0.04d	1.58 ± 0.02b	2.22 ± 0.03a	1.51 ± 0.03	<0.0001
Energy dissipated as heat	ΦNPQ	0.21 ± 0.01e	0.26 ± 0.01c	0.23 ± 0.01d	0.28 ± 0.01b	0.33 ± 0.01a	0.27 ± 0.01	<0.0001
Unregulated energy	ΦNO	0.23 ± 0.01a	0.21 ± 0.01c	0.22 ± 0.01b	0.21 ± 0.01c	0.21 ± 0.01d	0.21 ± 0.01	<0.0001
Total active PSI centers	PSI_ac_	1.83 ± 0.09a	1.59 ± 0.06b	1.84 ± 0.07a	1.58 ± 0.05b	1.28 ± 0.07c	1.59 ± 0.03	<0.0001
Fraction of open PSI centers	PSI_oc_	0.32 ± 0.02a	0.26 ± 0.01b	0.31 ± 0.01a	0.27 ± 0.01b	0.22 ± 0.01c	0.27 ± 0.01	<0.0001
Over-reduced PSI	PSI_or_	0.36 ± 0.03b	0.41 ± 0.02b	0.35 ± 0.03b	0.41 ± 0.02b	0.47 ± 0.03a	0.41 ± 0.01	0.0153
Fraction of oxidized PSI centers	PSI_ox_	0.31 ± 0.02a	0.29 ± 0.01a	0.31 ± 0.02a	0.31 ± 0.01a	0.28 ± 0.02a	0.32 ± 0.01	
Electron transfer amplitude of P700 during the light-dark transition	P700_DIRK_	0.27 ± 0.01c	0.31 ± 0.01b	0.29 ± 0.01b	0.34 ± 0.01a	0.35 ± 0.01a	0.32 ± 0.01	<0.0001
Electron transfer constant	_k_P700	186.71 ± 14.34a	136.69 ± 9.41b	175.73 ± 11.95a	189.38 ± 8.36a	172.77 ± 10.71a	171.01 ± 4.76	0.0008
Electron transfer lifetime of P700	_t_P700	0.56 ± 0.01a	0.55 ± 0.01a	0.55 ± 0.01a	0.54 ± 0.01b	0.54 ± 0.01b	0.54 ± 0.01	0.0019
Initial velocity of electron transfer at P700 in steady state	P700_i_	0.07 ± 0.01b	0.07 ± 0.01b	0.07 ± 0.01a	0.08 ± 0.01a	0.08 ± 0.01a	0.07 ± 0.01	0.0002
Chronic photoinhibition	PI_Chr_	16.33 ± 0.41d	18.07 ± 0.27c	16.61 ± 0.34d	19.15 ± 0.24b	20.28 ± 0.32a	18.42 ± 0.15	<0.0001
Dynamic photoinhibition	PI_Dyn_	6.17 ± 0.48d	10.63 ± 0.31b	9.89 ± 0.41c	11.21 ± 0.28b	15.74 ± 0.36a	11.19 ± 0.21	<0.0001
Total photoinhibition	PI_Total_	22.47 ± 0.37e	28.67 ± 0.24c	26.47 ± 0.31d	30.33 ± 0.22b	36.45 ± 0.28a	29.58 ± 0.25	<0.0001
Stress tolerance index	STI	0.61 ± 0.01a	0.60 ± 0.01a	0.56 ± 0.01b	0.54 ± 0.01c	0.54 ± 0.01c	0.57 ± 0.01	<0.0001

Mean ± Standard error (SE). ^a, b, c:^ Averages with a letter in common between rows are not significantly different at 5% probability.

The data presented represents the average value of the variables in the different accessions that made up each type. The mean for each accession was obtained by correcting for the effect of different random factors such as i. plant growth conditions (field conditions, greenhouse conditions), ii. water supply level (well-watered conditions, water-stressed conditions), iii. time of day (predawn, midday), iv. day of measurement (1, 2, 3, 4), and v. replication (1, 2, 3).

(i) **Highly adaptive** (n = 34; 10.5% of the total accessions evaluated). The mechanisms of this group differ from the others by having a higher relative chlorophyll content (RC) and greater leaf thickness (LTh). This allows for superior photosynthetic performance that is attributed to the efficient use of energy absorbed at the electron transport level in the thylakoids, which is mainly directed to a higher quantum yield of PSII (ΦII and F_v_/F_m_), lower energy dissipation as heat (ΦNPQ and NPQ_t_), high linear electron flow (LEF), high fraction of PSI centers (PSI_oc_) and PSII in the open state (qL), and improved electron transfer time (_t_P700) (Table 1). These photosynthetically adaptive characteristics allow this group to maintain photosynthetic activity showing ability to recover from chronic photoinhibition and not increase total photoinhibition (Table 1). Photosynthetic antenna complexes capture light energy for photosynthesis, but under excess light conditions, they play a crucial role in preventing photoinhibition. While photosynthetic antenna complexes can be damaged by high light, in this first group of highly adaptive tepary bean accessions, they act as “safety fuses” for the more sensitive PSI and PS II reaction centers. Through mechanisms like NPQ (especially qE), the antenna system dissipates excess energy as heat, preventing the formation of damaging ROS and protecting the catalytic core of the photosystems from irreversible damage.
(ii) **Moderately adaptive** (n = 49; 15.1% of the total accessions evaluated). This group presented the highest gH+ and vH+ values, in addition to more negative LTD values (i.e., the leaf is cooler than the ambient temperature) (Table 1). By allowing to maintain photosynthetic functioning, this group differs from the first group of highly adaptive by exhibiting an increase in energy dissipation in the form of heat (ΦNPQ and NPQ_t_), without affecting the allocation of energy to PSII (ΦII and F_v_/F_m_) (Table 1). Likewise, a higher fraction of active (PSI_ac_) and oxidized (PSI_ox_) centers is presented. With the adjustments made by this second group, no damage to the photosynthetic apparatus was detected, allowing them to recover from dynamic photoinhibition without presenting chronic photoinhibition (Table 1).
(iii) **Slightly adaptive** (n = 79; 24.4% of the total accessions evaluated). It was found that to cope with the photoinhibitory stress condition this third group of slightly adaptive tepary bean accessions increased energy dissipation in the form of heat (ΦNPQ and NPQt) (Table 1), with a significant reduction in PSII efficiency (F_v_/F_m_) as well as the rate and conductance in the thylakoid membrane proton flux begins to be affected (gH+ and νH+) along with decreases in LEF (Table 1).
(iv) **Poorly adaptive** (n = 100; 30.9% of total accessions evaluated). The mechanisms of this fourth group are centered on a greater availability of protons within the thylakoid membrane (gH+), with increases in ECS_t_, as well as a higher fraction of oxidized centers (PSI_ox_) and higher values in _k_P700 and P700_i_ (Table 1). The allocation of energy dissipated as heat (ΦNPQ and NPQ_t_) is increased, with lower allocations in undissipated energy (ΦNO) (Table 1). These photosynthetic adjustments made by this typology are not enough and it starts to reduce photosynthetic efficiency (F_v_/F_m_ and ΦII), keeping these accessions under dynamic photoinhibition state without reaching chronic photoinhibition (Table 1).
(v) **Very poorly adaptive** (n = 61; 18.8% of the total accessions evaluated). The photosynthetic adjustments made by this fifth group of tepary bean accessions are not adequate to maintain photosynthetic efficiency without any effect. The main characteristics of this typology include lower relative chlorophyll content (RC), higher leaf temperature (LT) and lower leaf thickness (LTh) (Table 1). These characteristics are related to higher energy expenditure (lower LTD) and poorer ability to recover from higher NPQ and NPQ_t_ and lower values of ΦII and F_v_/F_m_. This translates into poor adaptation to stress leading to reduced photosynthetic efficiency, and these accessions fail to regulate energy dissipation and fluorescence emission. These conditions induce physical damage to the photosynthetic antenna complexes and lead to chronic photoinhibition. The photoprotective mechanisms of this fifth group of tepary bean accessions are not efficient enough due to an excess of excitation energy.

**Fig 2:**
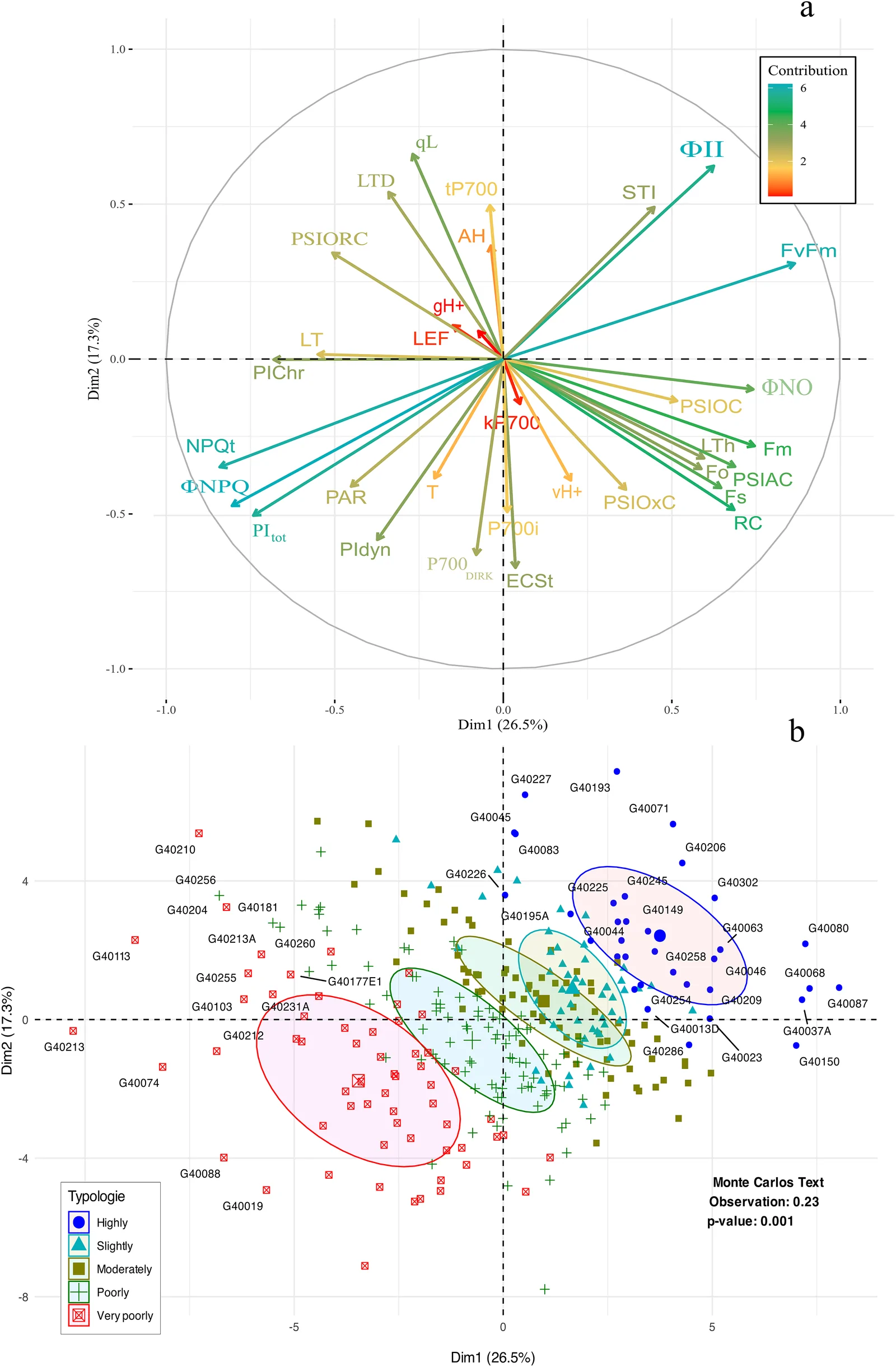
Projection of principal component analysis (PCA) of photosynthetic variables evaluated in different tepary bean accessions under acid soil and high temperature stress conditions in the Colombian Amazon: (a) correlation circle between the different physiological variables; (b) typologies of different tepary bean accessions. The gradient from green to red indicates a level of contribution from higher to lower. RC: Relative chlorophyll content, LT: Leaf temperature, LTD: Leaf temperature difference, LTh: Leaf thickness, ECSt: Maximum electrochromic displacement amplitude (x 1000), gH + : Proton conductance of ATP synthases in the thylakoid membrane, vH + : Rate of proton conductance of ATP synthases in the thylakoid membrane, F_m_: Maximum fluorescence, F_o_: Minimum fluorescence, F_s_: Steady-state fluorescence, F_v_/F_m_: PSII photochemical efficiency, ΦII: Fraction of energy devoted to photosystem II, LEF: Linear electron flow, qL: Fraction of open PSII reaction centers, NPQ_t_: Total energy dissipated as heat, ΦNPQ: Energy dissipated as heat, ΦNO: Unregulated energy, PSI_ac_: Total active PSI centers, PSI_oc_: Fraction of open PSI centers, PSI_or_: Over-reduced PSI, PSI_ox_: Fraction of oxidized PSI centers, P700_DIRK_: Electron transfer amplitude of P700 during the light-dark transition, _k_P700: Electron transfer constant, _t_P700: Electron transfer lifetime of P700, P700_i_: Initial velocity of electron transfer at P700 in steady state, PI_Chr_: Chronic photoinhibition, PI_Dyn_: Dynamic photoinhibition, PI_Total_: Total photoinhibition, STI: Stress tolerance index.

When relating each typology to the photosynthetic variables studied, it was observed that tepary bean accessions belonging to the highly adaptive typology exhibited a greater fraction of energy dedicated to the operation of the photosynthetic machinery (ΦII) as well as higher photosynthetic efficiency of PSII (F_v_/F_m_). Additionally, the open and active centers of PSI were more abundant in this typology. All of this translated into a greater adaptive capacity, measured through the STI value (Fig 2a). On the other hand, the typology of accessions with very low level of adaptation was associated with a greater fraction of energy dissipated as heat (ΦNPQ, NPQ_t_) and higher photoinhibition (PI_Total_) (Fig 2a). The analysis of the variance explained by the five typologies of the tepary bean accessions revealed that they account for 23% of the total variance (P < 0.001), distinguishing accessions from lower to higher level of adaptation to acidic soil and high temperature stress conditions in the Amazon, following a gradient from left to right in Fig 2b.

### 3.2. Adaptation mechanisms of tepary bean accession types based on their photosynthetic response

**Fig 3:**
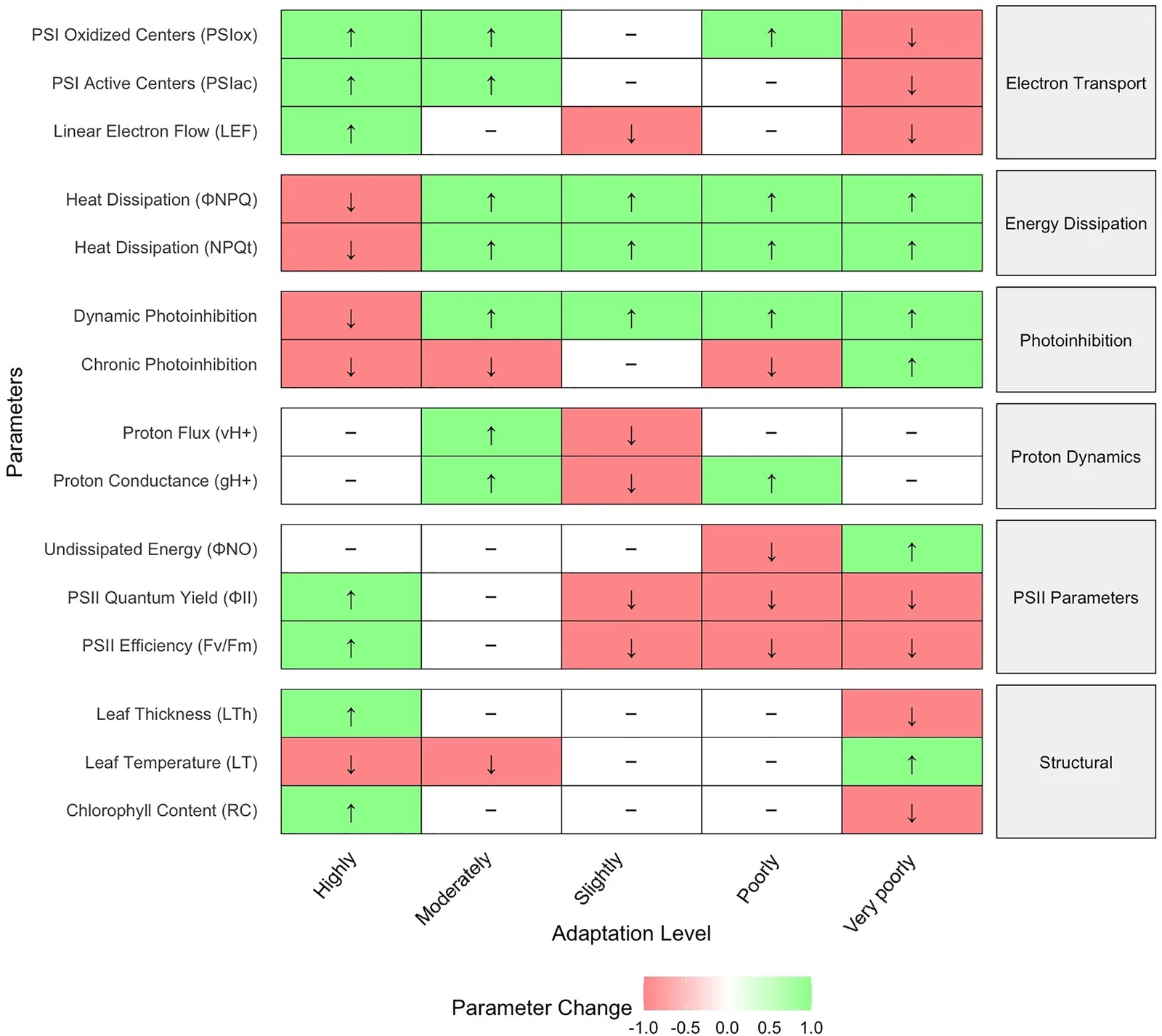
Comparison of photosynthetic adaptation mechanisms among tepary bean accessions. Green with upward arrows (↑) indicates significant increases in measured parameters, while red with downward arrows (↓) indicates significant decreases. Dashes (-) represent cases where no significant changes were observed in the evaluated parameters. The intensity of these changes is quantified on a red-to-green scale ranging from −1.0 to 1.0.

A detailed comparison of photosynthetic adaptation mechanisms in different accessions of tepary bean revealed significant patterns in multiple aspects of its physiology (Fig 3). Highly adaptive accessions demonstrate distinctive characteristics, including higher chlorophyll content, greater leaf thickness, and lower leaf temperature, suggesting better structural adaptation. These accessions also exhibit better photosystem II (PSII) efficiency, evidenced by superior F_v_/F_m_ values and quantum yield, which progressively decrease in less adapted accessions. Interestingly, an inverse pattern is observed in energy dissipation, where highly adaptive accessions show lower heat dissipation (ΦNPQ and NPQ_t_), while less adaptive ones present higher values of these parameters. Regarding proton dynamics, moderately adaptive accessions stand out for their higher conductance and proton flux, characteristics that are reduced in slightly adaptive accessions. Electron transport also shows notable differences, with highly adaptive accessions presenting greater linear electron flow and more active and oxidized PSI centers, while less adaptive accessions show a reduction in these parameters. Photoinhibition, both dynamic and chronic, tends to be lower in highly adaptive accessions, although chronic photoinhibition shows a more variable pattern. Together, these results indicate that highly adaptive accessions have developed more efficient mechanisms to handle environmental stress while maintaining high photosynthetic productivity, demonstrating better structural photosynthetic capacity, higher PSII efficiency, less need for energy dissipation mechanisms, more efficient electron transport, and better protection against photoinhibition.

**Fig 4:**
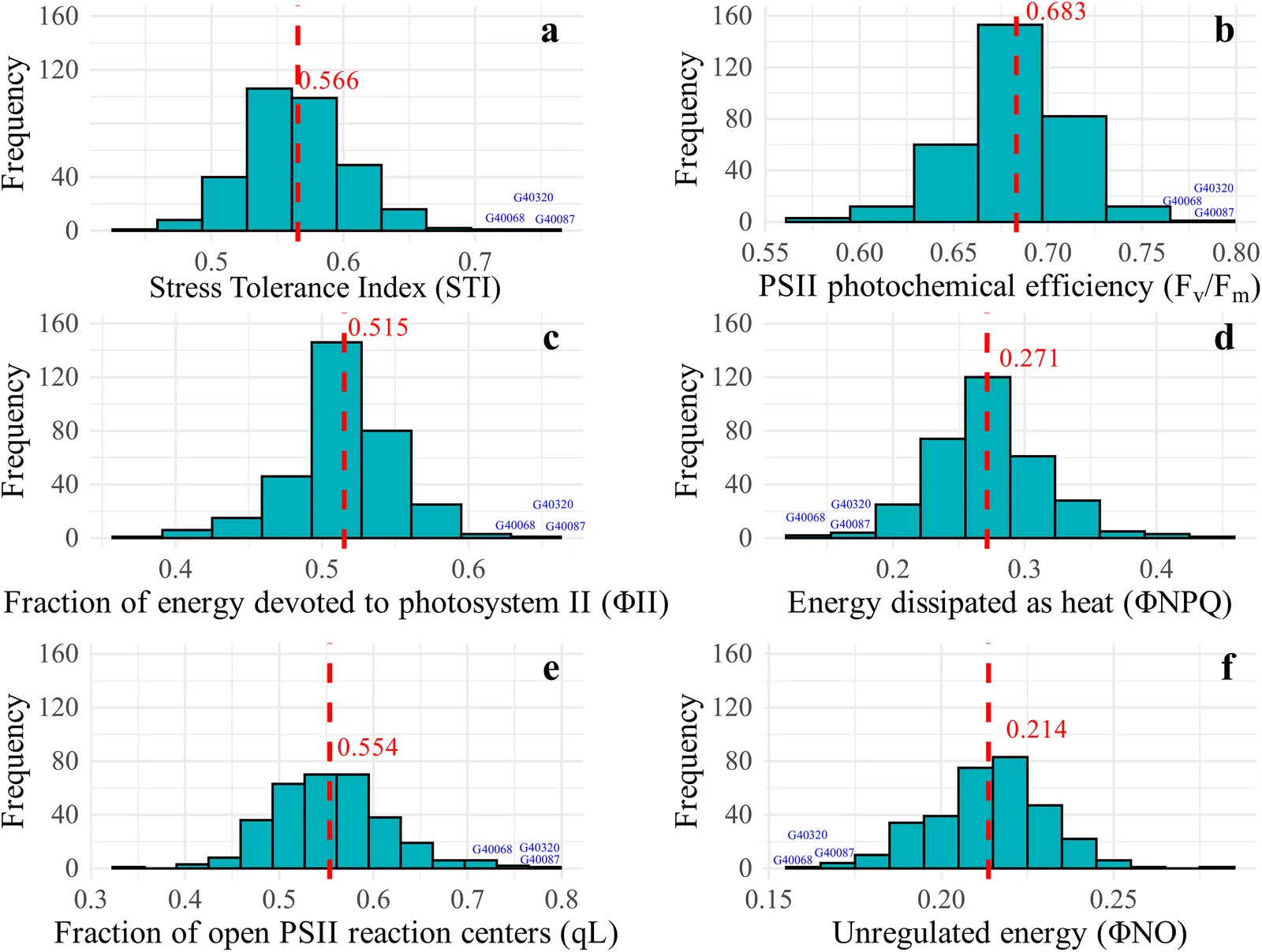
Frequency distribution of photosynthetic parameters of tepary bean accessions under acid soil and high temperature stress conditions. The red line in the middle indicates the mean value.

Fig 4 presents a detailed analysis of various photosynthetic parameters for different tepary bean accessions. The STI (Fig 4a) shows a mean value of 0.566, with a normal distribution suggesting a variable stress response among the evaluated genotypes. The photochemical efficiency of PSII (F_v_/F_m_, Fig 4b), with a mean value of 0.683, indicates that plants are experiencing some level of stress, as optimal values in healthy plants typically approach 0.83. The fraction of energy allocated to photosystem II (ΦII, Fig 4c) presents a mean value of 0.515, reflecting the proportion of energy effectively used in photosynthesis, with a symmetrical distribution suggesting a relatively uniform response among genotypes. The energy dissipated as heat (ΦNPQ, Fig 4d), with a mean value of 0.271, represents the photoprotection mechanisms that are being activated in response to stress. The fraction of open PSII reaction centers (qL, Fig 4e) shows a mean value of 0.554, indicating the availability of reaction centers for photosynthesis, with wide variation among genotypes. Finally, the non-regulated energy (ΦNO, Fig 4f), with a mean value of 0.214, represents uncontrolled energy losses, where lower values are preferable for better photosynthetic efficiency. The location of the three tepary bean accessions (G40320, G40068, G40087) within each figure represents the physiologically superior response values.

**Fig 5:**
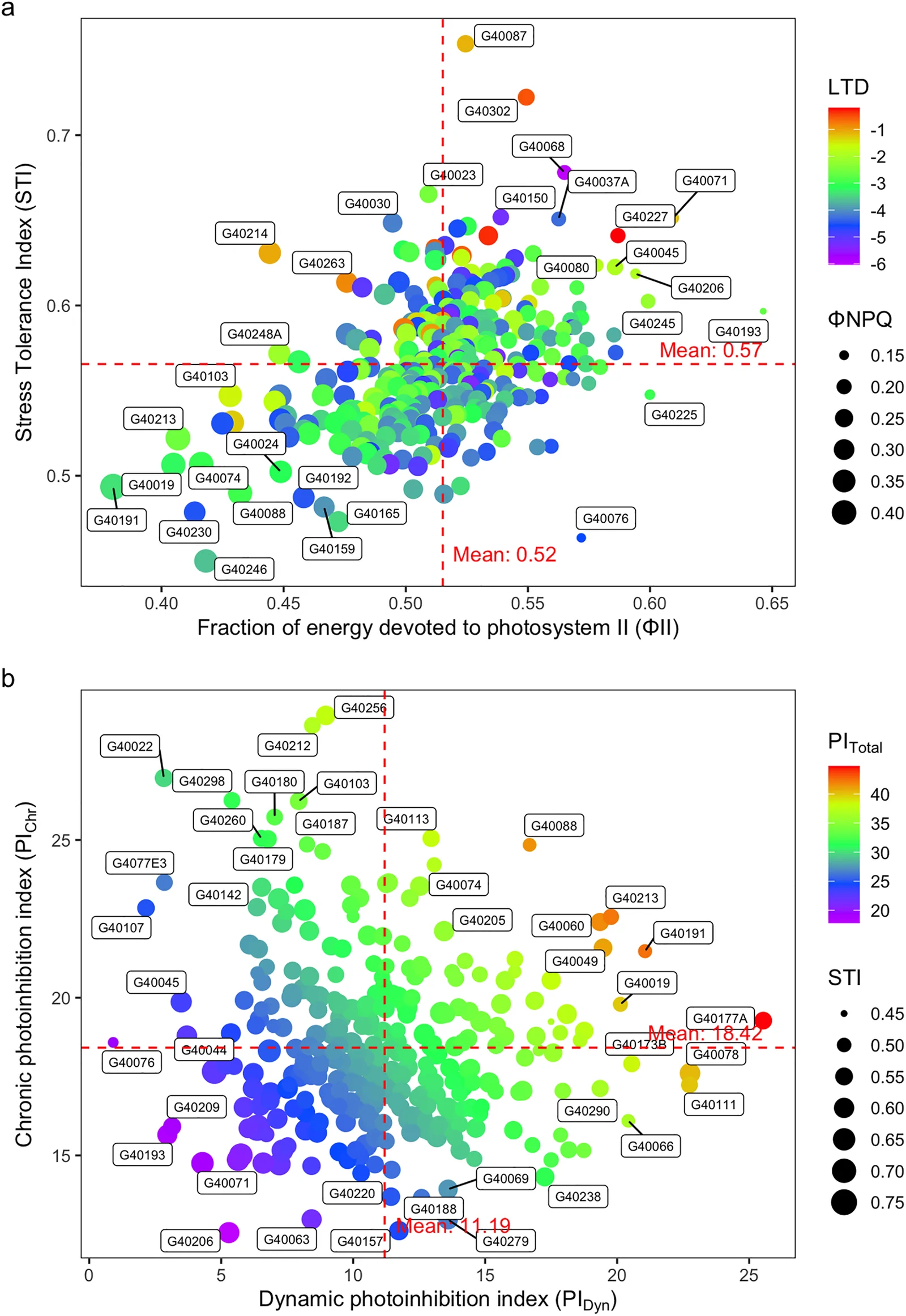
Relationship between different photosynthetic variables: a. leaf temperature difference (LTD) gradient (change from red to purple color from lowest to highest value) and the magnitude of energy dissipated as heat (ΦNPQ, circle size) as a function of the dependent and independent variables; b: Total photoinhibition gradient (PI_Total_) (change from red to violet color from highest to lowest value) and stress tolerance index (STI, circle size) based on the dependent and independent variables. The dotted lines on the axes correspond to the means of the dependent and independent variables.

The STI values ranged between 0.45 and 0.75, while ΦII values ranged between 0.40 and 0.65 (Fig 5a). A positive correlation between STI and ΦII (r = 0.78, P < 0.05) was observed, suggesting that accessions with higher levels of stress tolerance maintain better photosynthetic efficiency. Genotypes G40087 and G40302 exhibited higher STI values (0.75 and 0.72, respectively). In contrast, accessions G40246, G40076, and G40165 showed lower STI values (below 0.50), indicating lower level of stress tolerance. The negative relationship between STI and LTD suggests that more stress tolerant genotypes maintain better thermal regulation, while the negative correlation with ΦNPQ indicates greater efficiency in light energy use. The negative LTD values, varying between −6 and −1, indicate that leaves maintain temperatures below the ambient temperature, and this characteristic of leaf cooling was also observed with accessions that exhibited low STI values. ΦNPQ, which ranges between 0.15 and 0.40, shows that genotypes with higher STI values require less dissipation of excess energy, suggesting better stress adaptation.

Fig 5b shows significant patterns in the stress response of various plant genotypes, where chronic (PI_Chr_), dynamic (PI_Dyn_), and total (PI_Total_) photoinhibition indices were evaluated along with the STI. Results show that PI_Chr_ ranged between 12.55 and 28.94, while PI_Dyn_ varied from 0.91 to 25.53, resulting in a PI_Total_ spanning from 17.81 to 44.78, with STI values between 0.45 and 0.75. Among the accessions most notable for their stress tolerance, G40087, G40302, and G40068 stand out, with STI values of 0.754, 0.722 and 0.678, respectively, while three accessions G40246, G40076 and G40165 showed lower STI values of 0.450, 0.464 and 0.473, respectively. A positive correlation between PI_Chr_ and PI_Total_ was observed, with notable variability in PI_Dyn_ among different genotypes. The distribution of accessions shows a tendency to cluster at intermediate STI values (0.55–0.65), with clear differentiation between the most tolerant and susceptible genotypes. Genotypes exhibiting STI values above 0.70 represent valuable tepary bean genetic resources for bean breeding programs, while the variability observed in PI_Dyn_ suggests the existence of different adaptive strategies to tolerate acid soil and high temperature stress. The close relationship observed between PI_Chr_ and STI emphasizes the relevance of chronic photoinhibition as a crucial component in determining the level of stress tolerance among these tepary bean genetic resources.

### 3.3. Relationships between photosynthetic variables under conditions of combined stress

**Fig 6:**
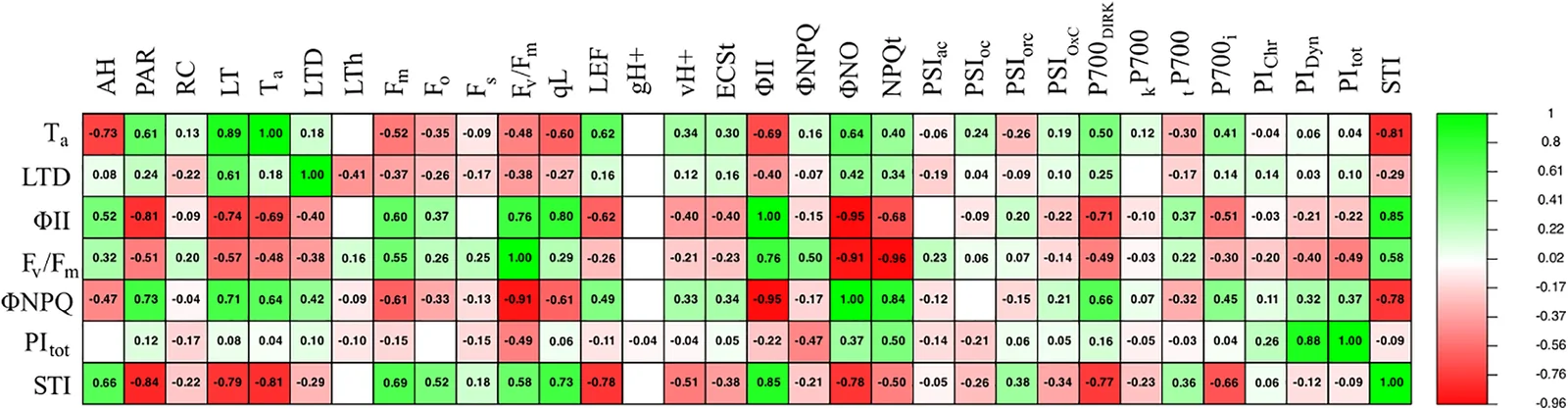
Correlation coefficients between different photosynthetic variables of tepary bean accessions under combined stress conditions of acid soils and high temperatures. A gradient from red to green indicates positive and negative correlations, respectively. Cells without color mean that there was no statistically significant correlation (P < 0.05). T_a_: ambient temperature; LTD: leaf temperature difference; ΦII: fraction of energy devoted to photosystem II; F_v_/F_m_: PSII photochemical efficiency; ΦNPQ: energy dissipated as heat; PI_Total_: total photoinhibition; STI: stress tolerance index; AH: ambient humidity; PAR: photosynthetically active radiation; RC: relative chlorophyll content; LT: leaf temperature; LTh: leaf thickness; F_m_: maximum fluorescence; F_o_: minimum fluorescence; F_s_: steady-state fluorescence; qL: fraction of open PSII reaction centers; LEF: linear electron flow; gH + : proton conductance of ATP synthases in the thylakoid membrane; vH + : rate proton conductance of ATP synthases in the thylakoid membrane; ECS_t_: maximum electrochromic displacement amplitude; ΦNO: unregulated energy; NPQ_t_: total energy dissipated as heat; different states of photosystem I (PSI) reaction centers: i. PSI_act_: active ii. PSI_ox_: oxidized, iii. PSI_open_: open state, iv. PSI_or_: over-reduced, P700_DIRK_: electron transfer amplitude of P700 during the light-dark transition, _k_P700: electron transfer constant, _t_P700: electron transfer lifetime of P700, P700_i_: initial velocity of electron transfer at P700 in steady state, PI_Chr_: chronic photoinhibition, and PI_Dyn_: dynamic photoinhibition.

At a general level, results presented in Fig 6 from the correlation analysis showed significant (P < 0.001) relationships among different physiological characteristics. In particular, based on the relationships among the leaf temperature difference (LTD) as canopy cooling capacity, light energy utilization (ΦII, ΦNPQ, F_v_/F_m_), total photoinhibition (PI_Total_), and the stress level of the tepary bean accessions measured (Stress tolerance index, STI), it was found that T_a_ correlated positively with LT and ECS_t_ as well as on the increase of heat energy fraction (ΦNPQ and NPQ_t_) and the increase on dynamic (PI_Dyn_) and total (PI_Total_) photoinhibition (Fig 6). However, T_a_ impacted negatively with variables related to photosynthetic functioning including ΦII, F_v_/F_m_, F_o_, F_m_, PSI_orc_, and P700_t_ as well as with STI (Fig 6). With increasing T_a_, plants dissipated high temperature stress by increasing LTD, which had a small negative effect on STI, and the different variables related to chlorophyll fluorescence (F_o_, F_m_, F_s_, F_v_/F_m_, qL) as well as the fraction dedicated to the photosynthetic machinery (ΦII) (Fig 6). Likewise, LTD was found to correlate positively with LEF, vH + , ECS_t_ as well as with the fraction of energy dissipated as heat (ΦNPQ, NPQ_t_) and the photoinhibition parameters of chronic (PI_Chr_) and total (PI_Total_) (Fig 6). Both ΦII and F_v_/F_m_ had positive correlation with STI, however, these variables presented negative correlation with both T_a_ and LTD as well as with the fractions that dissipate energy as heat (ΦNPQ, NPQ_t_). All variables related to photoinhibition (PI_Dyn_, PI_Chr_, PI_Total_) were also negatively correlated with ΦII and F_v_/F_m_. The PI_Total_ variable that shows the incidence of high temperature on tepary bean accessions shows that this variable was positively correlated with PAR, NPQ_t_ and P700_DIRK_. Finally, the STI value was found to decrease with increasing T_a_, since it affects different variables related to chlorophyll *a* fluorescence (F_o_, F_m_, F_s_, F_v_/F_m_, qL) (Fig 6).

### 3.4. Sensitivity of the Stress Tolerance Index (STI) as a function of the physiological variables

**Fig 7:**
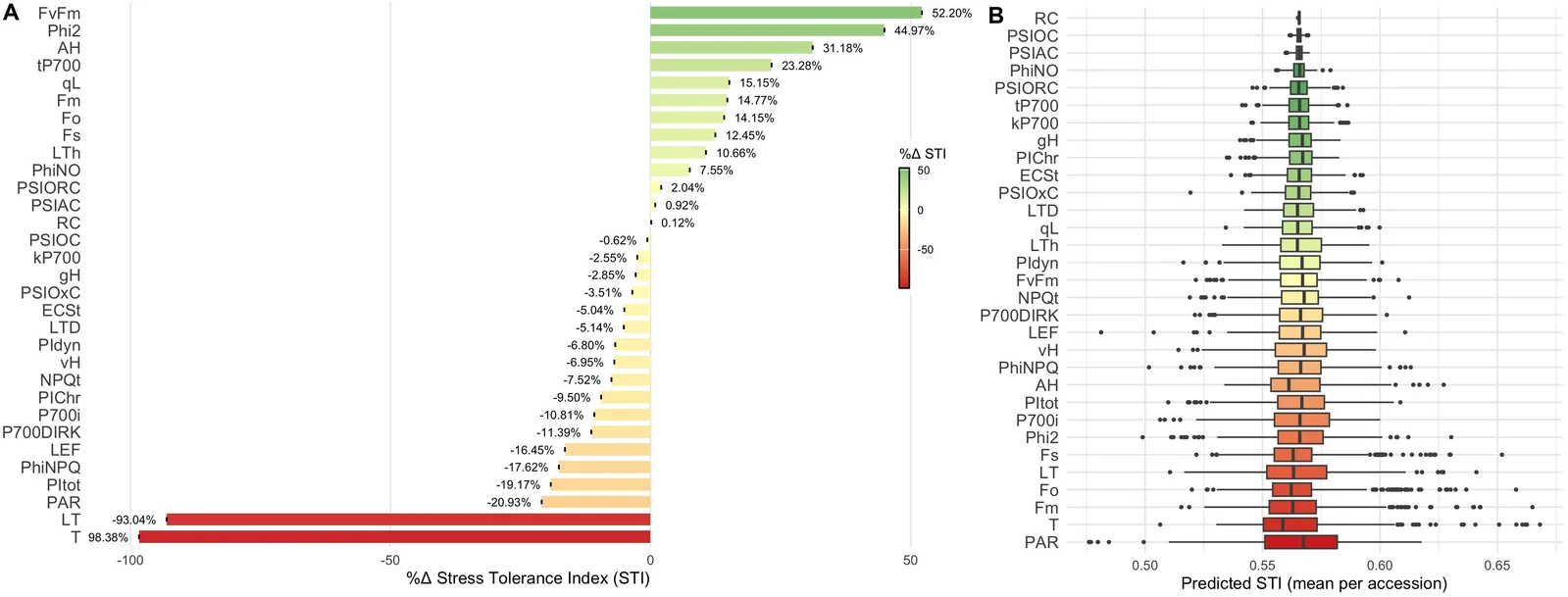
Change in the Stress Tolerance Index (STI) associated with a typical variation in each physiological variable. Positive values (to the right, in green tones) indicate that increasing that variable tends to increase STI; negative values (to the left, in red tones) indicate that increasing that variable tends to decrease STI. F_v_/F_m_: PSII photochemical efficiency; ΦII: fraction of energy devoted to photosystem II; AH: ambient humidity; _t_P700: electron transfer lifetime of P700; qL: fraction of open PSII reaction centers; F_m_: maximum fluorescence; F_o_: minimum fluorescence; F_s_: steady-state fluorescence; LTh: leaf thickness; ΦNO: unregulated energy; PSI_or_: over-reduced; PSI_act_: active; RC: relative chlorophyll content; PSI_open_: open state; _k_P700: electron transfer constant; gH + : proton conductance of ATP synthases in the thylakoid membrane; PSI_ox_: oxidized; ECS_t_: maximum electrochromic displacement amplitude; LTD: leaf temperature difference; PI_Dyn_: dynamic photoinhibition; vH + : rate proton conductance of ATP synthases in the thylakoid membrane; NPQ_t_: total energy dissipated as heat; PI_Chr_: chronic photoinhibition; P700_i_: initial velocity of electron transfer at P700 in steady state; P700_DIRK_: electron transfer amplitude of P700 during the light-dark transition; LEF: linear electron flow; ΦNPQ: energy dissipated as heat; PI_Total_: total photoinhibition; PAR: photosynthetically active radiation; LT: leaf temperature; T_a_: ambient temperature.

**Fig 8:**
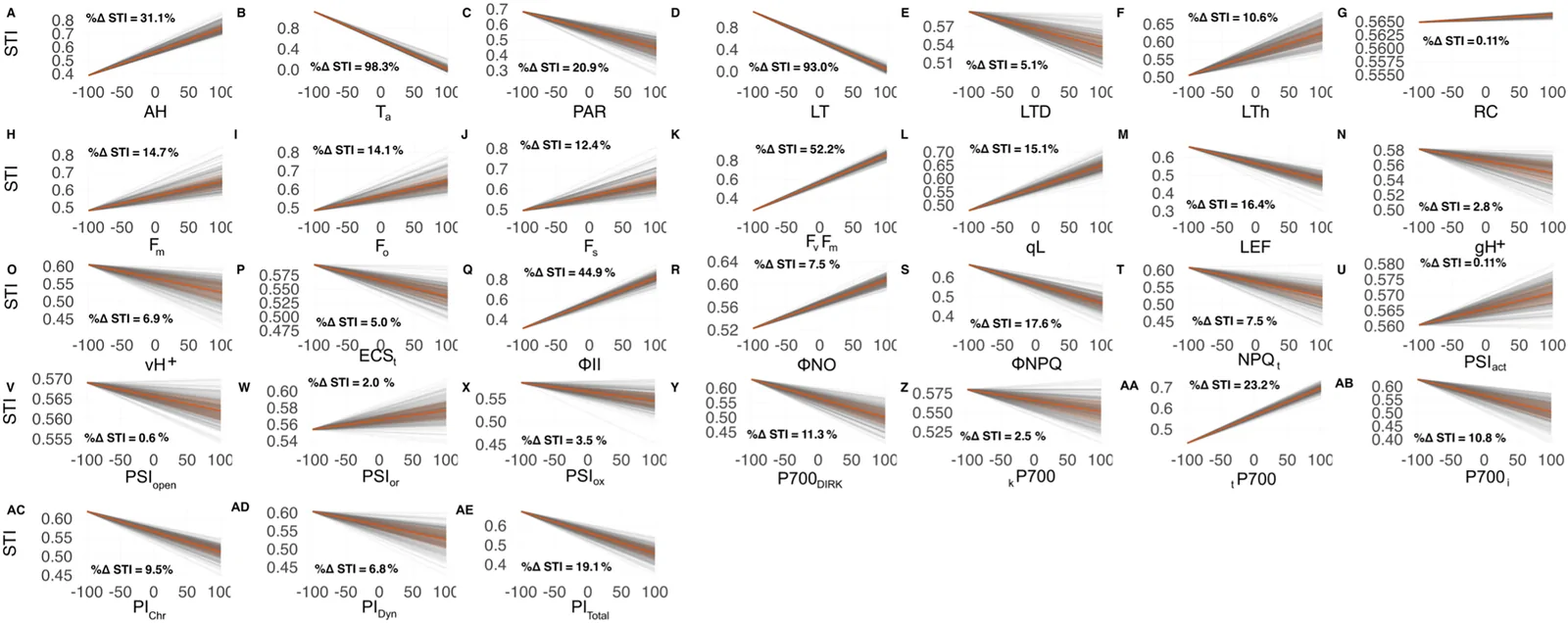
Sensitivity analysis of stress tolerance index (STI) in relation to the percentage change in physiological variables. A. AH: ambient humidity; B. T_a_: ambient temperature; C. PAR: photosynthetically active radiation; D. LT: leaf temperature; E. LTD: leaf temperature difference; F. LTh: leaf thickness; G. RC: relative chlorophyll content; H. F_m_: maximum fluorescence; I. F_o_: minimum fluorescence; J. F_s_: steady-state fluorescence; K. F_v_/F_m_: PSII photochemical efficiency; L. qL: fraction of open PSII reaction centers; M. LEF: linear electron flow; N. gH + : proton conductance of ATP synthases in the thylakoid membrane; O. vH + : rate proton conductance of ATP synthases in the thylakoid membrane; P. ECS_t_: maximum electrochromic displacement amplitude; Q. ΦII: fraction of energy devoted to photosystem II; R. ΦNO: unregulated energy; S. ΦNPQ: energy dissipated as heat; T. NPQ_t_: total energy dissipated as heat; different states of photosystem I (PSI) reaction centers; U. PSI_act_: active; V. PSI_open_: open state; W. PSI_or_: over-reduced; X. PSI_ox_: oxidized; Y. P700_DIRK_: electron transfer amplitude of P700 during the light-dark transition; Z. _k_P700: electron transfer constant; AA. _t_P700: electron transfer lifetime of P700; AB. P700_i_: initial velocity of electron transfer at P700 in steady state; AC. PI_Chr_: chronic photoinhibition; AD. PI_Dyn_: dynamic photoinhibition; AE. PI_Total_: total photoinhibition.

The results of the sensitivity analysis of the Stress Tolerance Index (STI) were derived from a fairly exhaustive process, which made it possible to understand the magnitude of the change (%, Fig 7A), the variability (dispersion of the means of the tepary accessions, Fig 7B), and the trend followed by each of the modeled curves for each tepary accession (Fig 8). The variables showing the largest positive effects on STI were F_v_/F_m_, ΦII and AH. Specifically, a typical change in F_v_/F_m_ was associated with an average increase of 52.2% in STI, representing the strongest positive association observed (Fig 7A). The ΦII also displayed a robust positive effect (+44.97%), while AH showed a substantial positive effect (+31.18%). Other physiological variables — _t_P700, qL, F_m_, F_o_, F_s_, LTh and ΦNO — exhibited moderate positive effects (approximately 2% to 24%), indicating that although they contribute to increases in STI, their impact is smaller than that of the three primary variables noted above (Fig 7A). Conversely, some variables were associated with substantially decreased values of STI. The most notable were T_a_ and LT, which showed unusually large average reductions (−98.38% and −93.04%, respectively). Other variables with moderate to large negative effects included PAR (−20.93%), PI_total_ (−19.17%), ΦNPQ (−17.62%) and LEF (−16.45%). Finally, a group of variables showed smaller negative effects, on the order of approximately −3% to −12% (e.g., P700_i_, P700_DIRK_, PI_Chr_, NPQ_t_, vH+ and PI_Dyn_) (Fig 7A). When analyzing the sensitivity level of each variable across different accessions (Fig 7B), a greater dispersion in the mean values of STI was observed for each accession in the variables PAR, T_a_, F_m_, and F_o_. In contrast, variables such as RC, PSI_or,_ PSI_act_, and ΦNO showed less variation in STI for each accession. Details on this trend and variability can be observed in Fig 8.

## 4. Discussion

The ambient conditions in the Colombian Amazon significantly influence the physiological and agronomic behavior of different tepary bean accessions [35], as well common bean breeding lines [28,53–55]. These previous studies have demonstrated the presence of both physiological and phenological traits and mechanisms that adapt to cope with the combined stress conditions of acidic soils and high temperatures.

This study resulted in classifying the 323 evaluated tepary bean accessions into five distinct typologies in terms of photosynthetic adaptation: (i) highly adaptive (10.5% of the total number of accessions evaluated), (ii) moderately adaptive (15.1%), (iii) slightly adaptive (24.4%), (iv) poorly adaptive (30.9%), and (v) very poorly adaptive (18.8%). The highly adaptive tepary bean accessions demonstrated outstanding photosynthetic adaptive mechanisms, as evidenced by a higher relative chlorophyll content reaching values between 45–52 and an increase in leaf thickness ranging between 280–320 μm, characteristics that enhanced their photosynthetic capacity. Some highly adaptive accessions included: G40087, G40302, G40068, G40023, G40150, G40071, G40037A and G40030. Some moderately adaptive accessions included: G40177D, G40271 and G40157.

In this study, a special effort was made in developing a stress tolerance index (STI) of tepary bean accessions as a helpful scorecard using PSII photoinhibition indices of chronic (PI_Chr_) and dynamic (PI_Dyn_) [23] that were based on the photochemical efficiency of PSII before predawn and at midday (F_v_/F_m_) under two plant growth environments (field, screenhouse) that generated contrasting ambient temperatures. Three tepary bean accessions (G40068, G40302, G40087) were identified, which, based on the STI considering various physiological response variables and PSII photoinhibition indices, demonstrated the ability to regulate and dissipate excess energy under stress conditions. Below, we describe the photosynthetic adjustment mechanisms and recovery capacity of the different tepary bean accessions in response to the combined stress of acidic soil and high temperature in the Colombian Amazon.

### 4.1. Energy dissipation mechanisms of tepary bean accessions in response to combined stress conditions

Tepary beans differ from common beans in their superior adaptation to high temperature and drought conditions [32,56]. Under combined stress of high temperature and acidic soil, certain accessions develop regulatory mechanisms to balance PSI and PSII performance [35]. Higher relative chlorophyll content and greater leaf thickness favor improved photochemical energy use (ΦII, F_v_/F_m_) and, under excess light, enable activation of energy dissipation mechanisms such as NPQ [57]. It is important to emphasize that the NPQ observed in the highly adaptive accessions acts primarily as a dynamic and reversible photoprotective mechanism — it dissipates excess energy as heat and reduces the likelihood of reactive oxygen species formation — and should not be confused with irreversible light damage. When NPQ increases together with full recovery of F_v_/F_m_ under low light or after night, we interpret this as an effective protection. In contrast, persistent reductions in F_v_/F_m_ over time indicate accumulated damage to PSII (chronic photoinhibition) [58]. Highly adaptive accessions (G40068, G40302, G40087, G40063, G40080, G40206, G40071, and G40041) excel in these photosynthetic adjustments, enabling faster recovery and avoiding chronic photoinhibition [59], demonstrating their superiority in photosynthetic apparatus performance [60].

Decreases in ECS_t_ (ATP synthase capacity) and LEF result from reduced intercellular CO_2_ concentration, limiting ATP synthase and NADPH/ATP utilization [61,62]. This PSII operational efficiency adjustment matches ATP and NADPH+ production with reduced CO_2_ assimilation [63] and metabolic consumption by Calvin cycle and anabolic pathways, addressing low thylakoid proton availability (gH+) [61,64]. Changes in ΦNPQ weakly affected LEF [65] because ΦNPQ competes with rapid photochemical excitation energy capture [46]. The LEF’s rate-limiting step occurs at cytochrome b6f complex level, controlled by lumen pH, so moderate ΦNPQ changes minimally affect PSII quantum efficiency and LEF, though energy loss patterns change [66]. The LEF serves as the key regulatory mechanism in photosynthetic adjustments, directly relating to CEF (cyclic electron flow) to balance ATP/NADPH+ and prevent chronic photoinhibition [67], which is typical of moderately adaptive tepary bean accessions such as G40275, G40282 and G40117.

As stress severity increased, open PSII reaction centers (qL) and leaf thickness decreased, particularly in poorly adaptive accessions. Water deficiency (phase 55 BBCH) and nutrient deficiency (acidic soil) increased light sensitivity and maintained CO_2_ limitation, triggering decreases in net CO_2_ assimilation, PSII redox state, ΦII and F_v_/F_m_ [64]. Decreased ΦII and F_v_/F_m_ associated with increased ΦNPQ, decreased ΦNO, and notable NPQ_t_ increase [68]. Increase of NPQ_t_ reduces damage to photosynthetic apparatus under prolonged stress conditions [44]. Along with chlorophyll content reduction, light-harvesting complexes dissociate from reaction centers as adaptive [69] or resilience response [70] to decrease stress damage [64]. CO_2_ fixation presents slower kinetics [71,72], decreasing ATP synthase capacity and NADPH+ utilization from LEF [61,62], causing transient NADP+ depletion [73] due to excessive PSI electron transport restriction [74]. These changes to photosynthetic apparatus due to stress severity trigger PSI photoinhibition [75], increasing total photoinhibition from combined stress impact on photosynthesis of very poorly adaptive tepary bean accessions.

### 4.2. Adjustments in PSII to maintain moderate photoinhibition and prevent PSI photoinhibition

In field conditions, plants are exposed to rapidly changing environmental conditions, especially light, which can vary in magnitude in less than a second [67]. Photoinhibition of the photosystems (PSI and PSII) occurs when the light energy absorbed by the pigments exceeds the capacity of the photosynthetic apparatus [62,76]. When the absorbed light exceeds the requirements for the photosynthetic process, the ΔpH generated by the CEF negatively regulates the activity of the *b*_*6*_*f* complex and controls the electron flow from PSII to PSI [77,78]. This optimizes the redox state of P700 in PSI and minimizes the production of ROS during photosynthesis [79]. We observed that a decrease in ΦII, an increase in NPQ_t_, and a reduction in qL were accompanied by a significant increase in ECS_t_, indicating a higher thylakoid proton motive force (*pmf*). However, the proton conductivity of the thylakoid (gH^+^) did not show a significant increase, only small differences compared to other groups, suggesting that the increase in *pmf* in the more sensitive accessions is not due to a slowdown in ATP synthase activity [80], but rather to elevated electron flow rate (vH^+^) in these accessions [81].

This mechanism has important implications for the energetic limitations of photosynthesis, especially in proton transport [82]. The two components of *pmf*, ΔpH (proton translocation by plastoquinone reduction and reoxidation) and Δψ (electric field of vectorial electron transfer across the thylakoid membrane) [47,83], affect the regulation of the photosynthetic process [82]. Both ΔpH and Δψ drive ATP synthesis from ADP and inorganic phosphate in the chloroplast ATP synthase, regulating the balance between efficient energy storage and light capture [47], preventing photodamage caused by recombination reactions in PSII, which explains the need for complex systems to balance ions in chloroplasts [77].

The highly adaptive tepary bean accessions show a regulatory mechanism, such as the P700 oxidation system, to oxidize P700 and protect PSI against ROS damage. It is believed that the ΔpH across the thylakoid membrane regulates PQH2 oxidation (plastoquinol accumulation) in *b*_*6*_*f*, inducing P700 oxidation, which limits electron transfer and prevents accumulation in PSI electron acceptors [84]. This promotes the induction of NPQ (NPQt), which dissipates energy as heat and downregulates electron transport through the b6f complex [85], contributing to P700 oxidation and protection of PSI. We emphasize that the NPQ described here is a regulatory process that temporarily reduces photochemical efficiency to prevent over-reduction of PSI acceptors and the consequent generation of ROS. Only when NPQ is not accompanied by recovery of parameters such as F_v_/F_m_ or qL during low-light periods or at night should it be considered insufficient and associated with accumulated light damage [80].

### 4.3. Recovery capacity of the photosynthetic apparatus and its relation to better energy use

To distinguish reversible protection from irreversible damage, we interpret NPQ together with predawn/night recovery of F_v_/F_m_ and with the dynamic (PI_Dyn_) and chronic (PI_Chr_) photoinhibition indices: high NPQ accompanied by recovery of F_v_/F_m_ and low PI_Chr_ indicates protection, whereas high NPQ without recovery and elevated PI_Chr_ indicates accumulated light damage. The STI developed as a helpful scorecard in this study revealed that high temperature has a marked influence on photosynthetic adaptation in highly adaptive accessions. This is based on the observation that accessions of poorly adaptive and very poorly adaptive groups begin to exhibit a deteriorated capacity for electron transport in the photosynthetic machinery, as evidenced by decreases in F_v_/F_m_, F_o_, F_m_, F_s_, and qL, resulting in photoinhibition [86,87]. These decreases suggest that PSII activity would be limited by an accumulation of electrons in quinone A (Q_A_), implying a blockage in electron transfer after plastoquinone reduction, possibly on the PSI acceptor side [47,54]. The excess of electrons from PSII to PSI can lead to the reduction of PSI acceptors and the production of superoxide anion radicals [88], which increases the reduced P700 centers, associated with high total photoinhibition, as observed in the very poorly adaptive group of tepary bean accessions [89].

We observed an increase in energy dissipation as heat (NPQ) that, in the better-adaptive accessions, was predominantly photoprotective and reversible and did not cause a sustained impairment of linear electron flow (LEF) [65,90]. However, in less-adaptive accessions we detected persistent decreases in F_v_/F_m_ and qL over time, indicating chronic photoinhibition or accumulated light damage. Therefore, we distinguish three scenarios: (i) an increase in NPQ with recovery of F_v_/F_m_, which indicates photoprotective NPQ; and (ii) a sustained decrease in F_v_/F_m_ and qL despite NPQ, which constitutes a sign of light damage and limited recovery capacity; and iii) the LEF rate-limiting step is not at light capture but at the cytochrome *b*_*6*_*f* complex, controlled by lumen pH. This means that changes observed in ΦNPQ, PSII quantum efficiency (ΦII), and LEF are not strongly affected, but the way energy is dissipated in the photosynthetic apparatus changes to maintain balanced functioning [47,91].

Some photosynthetic adaptive traits shown by the evaluated accessions included greater allocation in PSII photochemistry (ΦII and F_v_/F_m_), maintaining higher photosynthetic efficiency, even after being exposed to prolonged high temperature periods [92]. Three tepary bean accessions (G40068, G40087, and G40302) stood out by presenting a STI value of above 0.7, based on effective use of energy dissipation as heat (ΦNPQ), allowing them to minimize the impact on PSII, maintaining constant LEF, low PI_Dyn_, and controlled PI_Chr_. It is assumed that F_v_/F_m_ values without stress effect are around 0.83 in many species [87], and the above mentioned three accessions showed an average value of 0.73. However, F_v_/F_m_ value was higher before predawn than in the afternoon, with an average of 0.74 before predawn and 0.68 at midday, reflecting a greater electron transport capacity supported by high chlorophyll content and photosynthetic apparatus integrity [73,92,93]. The results of this study indicate that selection based on photosynthetic response mechanisms could be a valuable approach for bean breeding, especially under the specific conditions of the Amazon region (acidic soil and high temperature).

### 4.4. Sensitivity analysis of STI to physiological variables for identifying key functional drivers of photosynthetic adaptation

The data from sensitivity analysis indicate that recorded temperatures (T_a_ and LT) exhibit very high within‑accession variability relative to between‑accession variability, implying these measurements respond rapidly and heterogeneously to microenvironmental conditions and stomatal status. Physiologically, absolute leaf and air temperatures are therefore poor indicators of genetic differences among accessions in this experiment; instead, the air- leaf temperature differential (LTD = T_a_ - LT) is a more robust functional metric of thermoregulatory capacity: accessions with consistently less negative values of high LTD demonstrate effective transpirational cooling associated with high stomatal conductance and greater hydraulic capacity, promoting higher gas exchange and potential productivity, whereas accessions with more negative values of low LTD reflect more closed stomata or hydraulic limitation and a conservative strategy under water or thermal stress. Parameters quantifying energy capture and photosynthetic capacity — such as LEF and ΦII — display moderate between‑accession variation and are thus informative of functional differences in light energy utilization. Accessions with elevated LEF and ΦII under comparable conditions indicate greater capacity to route electrons toward carbon fixation and, consequently, higher potential productivity. However, canonical indicators of maximum PSII photochemical efficiency, like F_v_/F_m_, show high within‑accession variability, suggesting these measurements are strongly influenced by light history and dark‑adaptation protocol. Therefore, F_v_/F_m_ is only reliable for detecting chronic or sustained impairment when measurement protocols are strictly standardized or repeated measures are averaged.

Energy dissipation capacity via NPQ/ΦNPQ and NPQ_t_ exhibits a relatively favorable signal‑to‑noise ratio across accessions, enabling identification of photoprotective strategies. Accessions with high ΦNPQ and NPQ_t_ demonstrate a clear propensity for thermal dissipation of excess excitation and protection of the photosynthetic apparatus, characteristic of a conservative strategy that prioritizes redox stability and avoidance of photo‑oxidative damage over immediate carbon gain. Conversely, accessions combining high LEF with moderate ΦNPQ and low LTD suggest a productivity‑oriented strategy that maximizes capture at the expense of increased water use and heightened risk under drought or heat. PSI‑related parameters (P700_i_, P700_DIRK_, _k_P700, _t_P700), and the total performance index (PI_Total_) exhibit between‑accession variability and this is sufficient enough to consider them robust candidates for discriminating differences in photosystem balance management and protective pathways. Variation in P700 metrics reflects differences in the oxidation–reduction dynamics of PSI, cyclic electron flow capacity, and the ability to avoid over‑reduction when NADPH demand or downstream electron acceptor capacity is limited. Accessions with favorable P700_i_ and P700_DIRK_ and appropriate recovery kinetics are likely to manage irradiance fluctuations and abrupt changes in demand more effectively, thereby preserving photosystem integrity. Parameters linked to proton coupling and ATP synthesis (gH + , vH+) show between‑accession variation that may reflect differences in the capability to convert thylakoid proton motive force into ATP, impacting the energetic balance between light capture and biosynthetic demand. Accessions with higher proton conductance could sustain greater ATP demand during active photosynthesis, contributing to increased efficiency in the coupling between electron flux and carbon assimilation.

Integrating these physiological traits suggests three plausible functional syndromes among accessions: (i) a “productivity” syndrome characterized by low LTD, high LEF and ΦII, moderate ΦNPQ, and high PI_Total_, indicative of genotypes optimized for maximal energy capture with higher hydric cost; (ii) a “protection/conservation” syndrome with high LTD, high NPQ_t_/ΦNPQ, and reduced LEF, reflecting genotypes that prioritize dissipation and avoidance of over‑reduction under stress; and a (iii) “redox robustness/recovery” syndrome where robust PSI metrics and PI_Total_ indicate superior control of photosystem balance and recovery capacity under fluctuating light. Intermediate phenotypes may combine relatively high LEF with dynamic NPQ mechanisms, suggesting plastic genotypes capable of switching between performance and protection depending on environmental context. From an applied interpretative standpoint, PSI metrics and several electron transport indicators (P700_i_, P700_DIRK_, PI_Total_, LEF, ΦNPQ) are the most promising traits for detecting genetic differences in energy management across accessions in this dataset, whereas absolute thermal measures and F_v_/F_m_ require stricter experimental control or replicate averaging to be informative. Ultimately, detecting bona fide adaptive differentiation will require integrating LTD values with capture and dissipation metrics (LEF, ΦII, NPQ_t_/ΦNPQ) and accounting for environmental history and water status as covariates. This is because the combination of thermoregulation, electron transport capacity, and dissipation pathways underpin the functional trade‑offs that determine adaptive strategies across tepary bean accessions.

### 4.5. Advances in understanding the photosynthetic adaptation of tepary bean to acidic soil and high temperature stress in the Colombian Amazon

Many previous studies evaluated stress factors in isolation. For example, experiments under restricted water conditions documented integrated responses in vegetative growth, chlorophyll content, and osmotic adjustments that favor survival but focused solely on water stress [94]. A comparative study on water relations and the maintenance of water potential and photosynthesis between *P. vulgaris* and *P. acutifolius* was focused mainly under drought stress conditions [95], and the results indicated that *P. acutifolius* maintains higher saturated photosynthetic rates and better stomatal regulation under restricted water supply. Similarly, heat tolerance was studied in the absence of water stress [96], and other works analyzed heat responses or morpho‑anatomical changes separately [32]. Studies on yield and genetic composition have integrated multiple environments but remain limited to reduced combinations of factors in each experiment [97,98]. Therefore, the present study is valuable and novel because it combines chlorophyll fluorescence measurements (F_v_/F_m_, ΦII, ΦNPQ, NPQ_t_, qL, LEF, PSI parameters), environmental variables (T_a_, LT, PAR), and morphological variables (leaf thickness, relative chlorophyll content) under multiple conditions (field/screenhouse, well-watered/water-stressed, predawn/midday) to generate a robust STI as a scorecard that helps to classify tepary bean accessions based on their tolerance and adaptive mechanisms. This facilitates identification of potential parental materials (e.g., G40068, G40302, G40087) for breeding programs aimed at improving adaptation to warm climates and acidic soils.

This study provides significant evidence on photosynthetic adaptation and stress tolerance of various tepary bean accessions. A more robust experimental design was employed compared to Suárez et al. [35] and Suárez and Rao [36], including temporal repetitions (planting seasons), evaluations under different conditions (field and screenhouse), periods of maximum and minimum rainfall, and samplings at different times of day (predawn and midday). This process was repeated for three consecutive days in each condition, allowing data collection to determine PSII photoinhibition indices: chronic photoinhibition (PI_Chr_) and dynamic photoinhibition (PI_Dyn_) following Werner et al. [23] approach. Finally, these data were used to develop a stress tolerance index (STI). Three highly adaptive tepary bean accessions (G40068, G40302, and G40087) were identified, demonstrating superior photosynthetic efficiency by maintaining high F_v_/F_m_ levels and low chronic photoinhibition. These accessions showed better energy regulation under stress through higher chlorophyll content and thicker leaves, enabling efficient energy dissipation and rapid recovery from photoinhibition. Significant correlations between ambient temperature (T_a_) and leaf temperature difference (LTD) negatively impacted photosynthetic variables (ΦII, F_v_/F_m_). Five typologies in terms of photosynthetic adaptation were identified: highly adaptive, moderately adaptive, slightly adaptive, poorly adaptive, and very poorly adaptive. These findings provide valuable insights to bean breeding programs for selecting parental lines to develop climate-adaptive varieties for the Colombian Amazon.

Regarding the results presented by Suárez et al. [35], morpho-phenological and agronomic differences were evidenced, with *P. acutifoliu*s var. acutifolius cultivated showing a higher number of pods per plant, larger seeds, and a greater number of seeds per pod. Significant variations were also identified in root biomass, days to flowering and physiological maturity, specific leaf area, and stomatal density among different genetic resource types. In terms of grain yield, 6 cultivated accessions and 19 wild accessions of *P. acutifolius* var. *tenuifolius* stood out with yields exceeding 1,800 kg ha^−1^, suggesting their potential as parental lines to improve dry seed production under combined stress conditions. Additionally, Suárez et al. [35] demonstrated energy dissipation mechanisms, with photochemical quenching (qP) being higher in cultivated *P. acutifolius* var. acutifolius, while energy dissipation through non-photochemical quenching (NPQ) was higher in regressive and wild *tenuifolius* accessions. In the results presented by Suárez and Rao [36], cluster analysis and principal component analysis (PCA) were used and five typologies were identified: TASHT (tolerant to acidic soils and high temperatures), HCCL (high leaf cooling capacity), HLA (higher leaf angles), HLT (higher leaf temperatures), and LASHT (low adaptation to acidic soils and high temperatures), based on information collected during midday samplings with temporal repetition (two planting seasons). This same study highlighted that correlation analyses showed differences in the photosynthetic response between TASHT and LASHT typologies, emphasizing the importance of energy dissipation as heat and increase in water use efficiency.

Based on results from this study, tepary bean accession G40087 emerges as the most highly adaptive, with a STI value of 0.754, supported by a robust photosynthetic system characterized by the highest chlorophyll content (RC = 54.54) and optimal fluorescence values. Its PSII photochemical efficiency (F_v_/F_m_ = 0.722) indicates a healthy photosynthetic apparatus, while its high proton conductance (gH+ = 154.88) and maximum electrochromic displacement (ECS_t_ = 3.186) suggest an effective ATP synthase capacity to maintain energy balance. Meanwhile, G40068 with a STI value of 0.678 demonstrates a unique adaptation strategy, showing the highest photochemical efficiency (F_v_/F_m_ = 0.737) and the highest fraction of energy devoted to photosystem II (ΦII = 0.565). This accession is distinguished by requiring less energy dissipation as heat, as evidenced by its low ΦNPQ (0.197) and NPQ_t_ (0.678) values, along with the low value of total photoinhibition (PI_Total_ = 20.73). Its greater leaf thickness (LTh = 0.644) contributes to better light energy management and protection of the photosynthetic apparatus. Another accession G40302, with a STI value of 0.722, exhibits a different but equally effective adaptive strategy. It is characterized by maintaining the highest fraction of open PSII reaction centers (qL = 0.562) and the highest NPQ_t_ (1.077), suggesting a greater capacity to dissipate excess energy when necessary. The above mentioned three tepary bean accessions exhibit superior photosynthetic adaptation strategies to combined acid soil and high temperature stress: G40087 excels in robust photosynthetic machinery, G 40068 in efficient energy use with minimal dissipation, and G40302 in flexible stress response. These adaptive attributes to protect their photosynthetic apparatus make them valuable tepary bean genetic resources for bean breeding programs that are active in developing varieties suited to the acid soil and climatic conditions of the Colombian Amazon region.

## 5. Conclusions

Three stress tolerant tepary bean accessions were identified: G40068, G40302 and G40087, which demonstrated superior photosynthetic adaptation mechanisms. Among these, G40087 stood out with the highest STI value (0.754), while G40068 showed the highest value of photochemical efficiency (F_v_/F_m_ = 0.737), and G40302 exhibited the best capacity to maintain open PSII reaction centers (qL = 0.562). This study also contributed to classify the 323 evaluated tepary bean accessions into five distinct typologies in terms of photosynthetic adaptation: highly adaptive (10.5% of the total number of accessions evaluated), moderately adaptive (15.1%), slightly adaptive (24.4%), poorly adaptive (30.9%), and very poorly adaptive (18.8%). The highly adaptive tepary bean accessions demonstrated outstanding photosynthetic adaptive mechanisms, as evidenced by a higher relative chlorophyll content reaching values between 45−52 and an increase in leaf thickness ranging between 280−320 μm, characteristics that enhanced their photosynthetic capacity. The efficiency in energy dissipation towards PSII was reflected in a remarkable maximum photochemical efficiency of 0.737 and an effective quantum yield above 0.45 under stress conditions. Particularly noteworthy was the optimization in energy dissipation as heat, where ΦNPQ values remained in a favorable range of 0.28–0.35. This efficient regulation was complemented by an outstanding recovery capacity, as evidenced by chronic photoinhibition indices below 0.15 and a post-stress recovery rate exceeding 85% of initial photochemical efficiency after 24 hours, thus demonstrating remarkable resistance to chronic photoinhibition and superior photosynthetic adaptation to adverse environmental conditions. This study developed and validated the stress tolerance index (STI) as a helpful scorecard based on measurements of both chronic and dynamic photoinhibition. Higher temperature emerged as the main stress factor, to which tepary bean accessions showed different physiological adaptation mechanisms, including canopy temperature regulation, adjustments in photosynthetic efficiency, and heat energy dissipation mechanisms.

## Supporting information

S1 File Variables monitored in the different materials. (XLSX)
